# Type II restriction endonucleases—a historical perspective and more

**DOI:** 10.1093/nar/gku447

**Published:** 2014-06-26

**Authors:** Alfred Pingoud, Geoffrey G. Wilson, Wolfgang Wende

**Affiliations:** 1 Institute of Biochemistry, Justus-Liebig-University Giessen, Heinrich-Buff-Ring 58, D-35392 Giessen, Germany; 2 New England Biolabs Inc., 240 County Road, Ipswich, MA 01938-2723, USA

## Abstract

This article continues the series of Surveys and Summaries on restriction endonucleases (REases) begun this year in *Nucleic Acids Research*. Here we discuss ‘Type II’ REases, the kind used for DNA analysis and cloning. We focus on their biochemistry: what they are, what they do, and how they do it. Type II REases are produced by prokaryotes to combat bacteriophages. With extreme accuracy, each recognizes a particular sequence in double-stranded DNA and cleaves at a fixed position within or nearby. The discoveries of these enzymes in the 1970s, and of the uses to which they could be put, have since impacted every corner of the life sciences. They became the enabling tools of molecular biology, genetics and biotechnology, and made analysis at the most fundamental levels routine. Hundreds of different REases have been discovered and are available commercially. Their genes have been cloned, sequenced and overexpressed. Most have been characterized to some extent, but few have been studied in depth. Here, we describe the original discoveries in this field, and the properties of the first Type II REases investigated. We discuss the mechanisms of sequence recognition and catalysis, and the varied oligomeric modes in which Type II REases act. We describe the surprising heterogeneity revealed by comparisons of their sequences and structures.

## PROLOGUE

We wonder what Molecular Biology would look like today had Type II restriction enzymes not been discovered. Synthesized in bewildering variety by bacteria and archaea to combat viral infections, these enzymes allow unmanageable tangles of macromolecular DNA to be transformed with unsurpassable accuracy into convenient, gene-sized pieces, a necessary first step for characterizing genomes, sequencing genes, and assembling DNA into novel genetic arrangements. It seems unlikely that today's Biomedical Sciences and the Biotechnology industry would have developed without Type II restriction enzymes, and certainly not at the startling pace we have witnessed since their discovery only a few decades ago.

## INTRODUCTION

**Figure 1.**
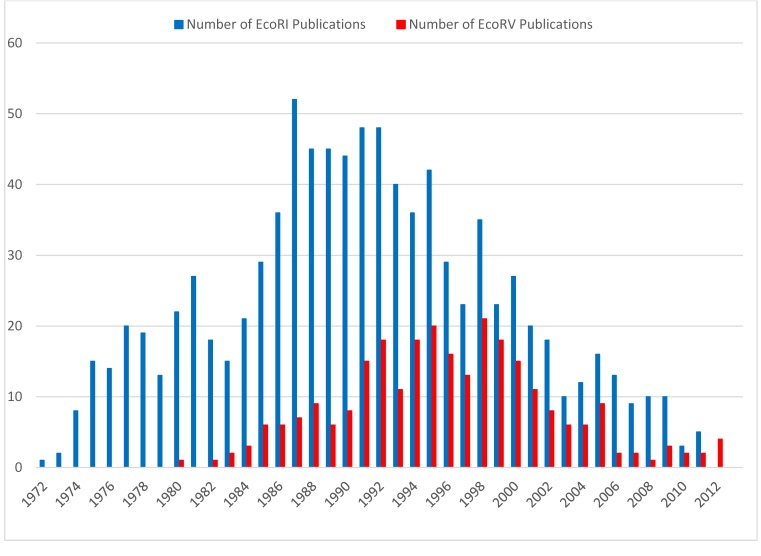
Number of publications for EcoRI and EcoRV per year from 1972 to 2012. Only publications are listed in which EcoRI and EcoRV are listed in the title. Source: REBASE (7).

Several reviews of restriction endonucleases (REases) have appeared as Surveys and Summaries in Nucleic Acids Research recently. These concerned the somewhat esoteric Type I (1), Type III (2) and Type IV (3) REases; highlights of half a century of REase research and discovery (4); and the connection between REases and genetic addiction systems (5). The present review focuses on the more familiar, Type II REases, the ‘work horses’ (6) of modern molecular biology, used daily in laboratories for DNA analysis and gene cloning. This review is partly historical, as were the others, and emphasizes the importance of the enzymes EcoRI and EcoRV, among the first REases discovered, and the two most thoroughly studied (Figure 1). It is also partly contemporary, and provides an up-to-date overview of the field, although one that is necessarily not comprehensive. Over 350 different Type II prototype REases are known, each unique in its biochemistry, and with its own story to tell. For most of these, anywhere from a few to over one hundred similar enzymes from sequenced organisms are known, some characterized but most putative. And REBASE (rebase.neb.com/rebase/rebase.html), the definitive source for information on REases and their companion proteins (7), lists over 8000 research publications in this field, too many by far to be discussed here. We apologize in advance for our omissions. For a broader review of Type II REases see Pingoud *et al.* (8). A comprehensive collection of reviews on REases has been published as a book: Pingoud (Ed.) REases (9). Two excellent additional reviews describe early work on Type II REases by Modrich & Roberts (10) and Roberts & Halford (11).

Following the original proposal by Smith and Nathans (12), restriction enzymes are named according to the taxonomy of the organism in which they were discovered. The first letter of the enzyme refers to the genus of the organism and the second and third to the species. This is followed by letters and/or numbers identifying the isolate. Roman numerals are used, finally, to specify different enzymes from the same organism. For example, the enzyme ‘HindIII’ was discovered in *Haemophilus influenzae*, serotype d, and is distinct from the HindI and HindII endonucleases also present in this bacterium. The DNA-methyltransferases (MTases) that accompany restriction enzymes are named in the same way, and given the prefix ‘M.’. When there is more than one MTase, they are prefixed ‘M1.’, ‘M2.’, if they are separate proteins and ‘M.’ or ‘M1∼M2.’ when they are joined. REases are designated explicitly by the prefix ‘R.’; this is usually omitted when there is no ambiguity. Enzymes in which restriction and modification activities occur in the same polypeptide chain are prefixed 'RM.' (e.g. RM.BcgI), which again is omitted when there is no ambiguity. Additional proteins are prefixed ‘V.’ (for Vsr endonucleases) and ‘C.’ (for control proteins). For example, the AciI R–M system, from *Arthrobacter citreus*, comprises AciI (or R.AciI), an REase; M1∼M2.AciI (or M.AciI), a composite, double MTase, and C.AciI, a control protein. REases that recognize the same DNA sequence, regardless of where they cut, are termed ‘isoschizomers’ (*iso* = equal; *skhizo* = split) (13). Isoschizomers that cut the same sequence at different positions are further termed ‘neoschizomers’ (*neo* = new) (14). Isoschizomers that cut at the same position are frequently, but not always, evolutionarily drifted versions of the same enzyme (e.g. BamHI and OkrAI). Invariably, neoschizomers are different enzymes altogether (e.g. EcoRII and MvaI).

Like the other types of restriction enzymes, Type II REases occur exclusively in unicellular microbial life forms—mainly bacteria and archaea (prokaryotes)—and are thought to function primarily to protect these cells from viruses and other infectious DNA molecules. A group of large viruses that infect the eukaryotic algae, *Chlorella*, also encode Type II REases (15,16) and DNA-methyltransferases (MTases; (17)). The genes for Type II REases occur mainly on chromosomes, and occasionally on transmissible elements such as plasmids, transposons and insertion sequences. They rarely occur on bacteriophages, although MTases sometimes do, as one of several forms of viral self-protection (18–20). In the discussions that follow, we refer to all of these sources loosely, as ‘prokaryotes’, or ‘microbes’. Type II REases are more heterogeneous than the other REase types in part because ‘Type II’ is a utilitarian classification, based on enzymatic behavior rather than phylogeny. Type II REases are a conglomeration of many different proteins that, by definition, have the common ability to cleave duplex DNA at a fixed position within, or close to, their recognition sequence. This cleavage generates reproducible DNA fragments, and predictable gel electrophoresis patterns, properties that have made these enzymes invaluable reagents for laboratory DNA manipulation and investigation. Almost all Type II REases require divalent cations—usually Mg^2+^—as essential components of their catalytic sites. Many can use Mn^2+^ in place of Mg^2+^, and a few can use a variety of cations including Co^2+^, Zn^2+^, Ni^2+^ and Cu^2+^ instead (21). Ca^2+^ ions usually, but not always, inhibit catalysis. A few REases require Zn^2+^ ions (e.g. BslI, PacI and DpnI (22–24)), or less often Fe^2+^ ions (e.g. NotI (25)), for incorporation into Cys4 structural motifs. And a diverse subclass that catalyze DNA methylation in addition to cleavage (the Type IIG enzymes, discussed later) require the cofactor S-adenosylmethionine (AdoMet or SAM), often for both activities. Much of what we know about Type II enzymes was discovered first with EcoRI and EcoRV. These REases are representative of the Type II**P** subclass that recognize **p**alindromic (symmetric) DNA sequences and generally act as homodimers or homotetramers. Type IIP REases are the most familiar, and the most diverse, of the several Type II subclasses (26), but as we describe later, by no means the only kind. See Roberts *et al.* (14) for the current classification of Type II REases.

In this review, we describe some of the progress that has been made elucidating the structures, functions and evolution of Type II REases in general, and of EcoRI and EcoRV in particular. We hope to make clear how research on Type II REases has advanced our understanding of protein–DNA interactions. We discuss how these proteins locate and recognize their target sequences in DNA, how they catalyze DNA strand cleavage, how they might have evolved, and finally, how some are being repurposed to perform novel reactions for genome editing applications and gene therapy.

### Discovery of the first Type IIP restriction enzymes

**Figure 2.**
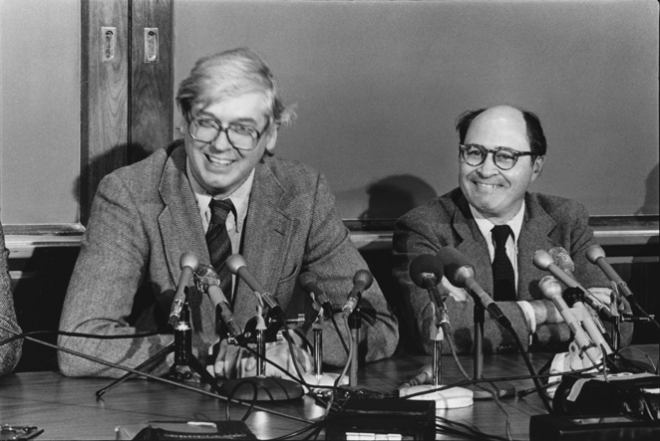
Hamilton Smith and Daniel Nathans at the Nobel Prize press conference, 12 October 1978 (reproduced with permission from Susie Fitzhugh). Original Repository: Alan Mason Chesney Medical Archives, Daniel Nathans Collection.

The first Type II REase discovered was HindII from the bacterium *Haemophilus influenzae* Rd. The event was described by Hamilton Smith (Figure 2) in his Nobel lecture, delivered on 8 December 1978:

‘In one such experiment we happened to use labeled DNA from phage P22, a bacterial virus I had worked with for several years before coming to Hopkins. To our surprise, we could not recover the foreign DNA from the cells. With Meselson's recent report in our minds, we immediately suspected that it might be undergoing restriction, and our experience with viscometry told us that this would be a good assay for such an activity. The following day, two viscometers were set up, one containing P22 DNA and the other *Haemophilus* DNA. Cell extract was added to each and we began quickly taking measurements. As the experiment progressed, we became increasingly excited as the viscosity of the Haemophilus DNA held steady while the P22 DNA viscosity fell. We were confident that we had discovered a new and highly active restriction enzyme. Furthermore, it appeared to require only Mg^2+^ as a cofactor, suggesting that it would prove to be a simpler enzyme than that from *E. coli* K or B. After several false starts and many tedious hours with our laborious, but sensitive viscometer assay, Wilcox and I succeeded in obtaining a purified preparation of the restriction enzyme. We next used sucrose gradient centrifugation to show that the purified enzyme selectively degraded duplex, but not single-stranded, P22 DNA to fragments averaging around 100 bp in length, while Haemophilus DNA present in the same reaction mixture was untouched. No free nucleotides were released during the reaction, nor could we detect any nicks in the DNA products. Thus, the enzyme was clearly an endonuclease that produced double-strand breaks and was specific for foreign DNA. Since the final (limit) digestion products of foreign DNA remained large, it seemed to us that cleavage must be site-specific. This proved to be case and we were able to demonstrate it directly by sequencing the termini of the cleavage fragments.’

### Isolation of Type II REases from bacterial extracts and their use for physical mapping of DNA

Early research into the phenomenon of restriction and modification (R–M) relied on measuring how well phage infected new bacterial hosts, an assay termed ‘efficiency of plating’ (eop) performed on lawns of bacteria growing in Petri dishes (27–29). Understanding of R–M leaped when biochemistry was brought to bear, and modification was shown to be the result of DNA methylation, and restriction the result of DNA-degradation (30). Initially, REase activities were measured by viscometry, but following the discovery of the ‘Type II’ (31) kind of REases that cleave DNA at fixed positions, further such enzymes were detected almost exclusively by assaying cell extracts for site-specific DNA-cleavage activity (13). This cleavage converts defined DNA molecules such as bacteriophage λ into a set of discrete fragments that produce a distinct banding pattern when electrophoresed through polyacrylamide (32), or agarose, gels (33,34); see, for example (35). Visualized by ethidium bromide staining of the fragments (34), gel electrophoresis in tubes, then vertical slabs, and finally submerged horizontal slabs, became a universal technique in molecular biology laboratories, culminating in the development of DNA fingerprinting (36).

HindII was the first Type II REase to be characterized (37,38) and used in this way (33), followed by EcoRI and EcoRII from *Escherichia coli* (39), and several others from *Haemophilus aegypticus* (40) and *H. parainfluenzae* (34,41). Interestingly, unbeknownst to Smith, the first preparations of HindII contained a second Type II REase, HindIII (42). Its presence would have interfered severely with analysis of the recognition sequence of HindII but for the good fortune that phage T7 DNA—the substrate used for this analysis—has no sites for the HindIII (43)! The pioneering work of Nathans *et al.* (Figure 2) (33,44–45), in which HindII was used to physically map the genome of the tumor virus SV40, stimulated the search for new REases with differing specificities. A prominent role in this endeavor, and ever since, was played by Rich Roberts, who early grasped the importance of these enzymes, and whose laboratory at Cold Spring Harbor served as a center for their discovery, characterization, cataloging and dissemination (13). By 1978, approximately 150 Type II REases with 50 different sequence specificities were known, including many ‘isoschizomers’ that recognize the same DNA sequence, and several ‘neoschizomers’ such as SmaI and XmaI that recognize the same sequence but cleave at different positions (46). Today, not counting putative enzymes, approximately 4000 Type II REases with over 350 different specificities have been identified (7).

Typical purification procedures for Type II enzymes started from a high-speed supernatant of a cell lysate, followed by removal of nucleic acids by streptomycin or polyethylene imine and several column chromatography steps, using typically phosphocellulose, DEAE-cellulose, hydroxyapatite, and gel filtration (13). Preparations were purified to the point they were free of interfering activities, but usually not to homogeneity. Their activity was (and still is today) usually given in arbitrary units, namely the amount of enzyme needed to completely digest 1 μg of λ DNA in 1 h at optimum temperature—usually 37°C. Because the intracellular concentration of Type II REases is usually low, often only a few milligrams could be isolated from kilogram amounts of wet cell paste following a tedious end lengthy isolation procedure.

### Sequence specificities of REases and the beginning of recombinant DNA research

Determining the recognition sequence of a Type II REase is a simple matter, today, but it was far from simple, initially. It required considerable experimental skill, knowledge and patience as even a glance at the seminal papers makes clear (38,47–48). The first recognition sequence to be determined, that of HindII, was found to be ambiguous (‘degenerate’) at the central base pair positions: 5′…GTPy|PuAC…3′ 3′…CAPu|PyTG….5′, or GTY|RAC for short (where Py and Y = C or T (pyrimidine); Pu and R = A or G (purine); and ‘|’ indicates the position of cleavage) (38). The next, for EcoRI, was unambiguous: 5′…G|AATTC…3′ 3′…CTTAA|G….5′, or G|AATTC (49). And the third, for EcoRII, had a different ambiguity, W (A or T; weak base-pairing), at the center: 5′…|CCAGG…3′ 3′…|GGTCC…5′, or |CCWGG (47,50). Phosphodiester bond cleavage in all three cases was found to generate 5′-phosphoryl and 3′-hydroxyl terminal groups. This has since been found to be true of all REases.

A striking feature of these three recognition sequences is their rotational symmetry. This symmetry, it was suggested (31), likely resulted from the subunit structure of the enzymes which interacted with the sequences in a symmetrical way. In confirmation, EcoRI was found to be composed of two identical subunits, and to cleave both strands of the DNA in one binding event, with no accumulation of an open circle (‘nicked’) intermediate (51). Later, kinetic experiments demonstrated that the two subunits cooperate in binding and cleaving the palindromic substrate (52). An important distinction between HindII and EcoRI is that cleavage by HindII is blunt, producing fragments with flush ends, whereas cleavage by EcoRI is staggered, producing fragments with 4-nucleotide single-stranded overhangs, 5′-pAATT…. Since these overhangs are complementary, and all fragments have the same overhangs, they ‘*…afford the possibility of reconstructing DNA molecules in vitro from any two DNA fragments generated by RI endonuclease digestion*’ (48). Mertz and Davis (53) came to the same conclusion: ‘*Therefore, any two DNA molecules with RI sites can be recombined at their restriction sites by the sequential action of RI endonuclease and DNA ligase to generate hybrid DNA molecules*’. It is fair to say that these insights heralded the start of recombinant DNA research (54) and genetic engineering (55) (see reflections by Berg and Mertz (56), and by Cohen (57)).

#### Effect of sequences flanking the recognition site on the cleavage activity of REases

Early studies on EcoRI focused on the cleavage of plasmid and phage DNA molecules. The rate at which EcoRI cleaved EcoRI sites was shown to depend upon flanking sequences (58–61). Later, this was systematically analyzed with synthetic oligonucleotides (62,63). Similar studies were carried out with other REases, including EcoRV (64). Not unexpectedly, it was found that flanking sequences in general modulate the thermodynamic and kinetic parameters of the interaction between REases and their targets. EcoRI, for example, interacts symmetrically with a minimum of 10 nucleotide pairs (65), which accounts in part for why it cleaves the 8 bp oligonucleotide, TGAATTCA, 200 times less efficiently than the equivalent natural site in SV40 DNA (66). The conformation of the DNA of the recognition sequence is also influenced by the surrounding sequence (67), which might also affect the rate of DNA cleavage by REases. Using a selection assay, variants of EcoRI were isolated that differed from the wild-type enzyme in their preference for flanking sequences (68). Similarly, EcoRV variants with different flanking sequence preferences could be engineered by a structure-guided design (69).

#### Star activity and the accuracy of REases

At low ionic strength and alkaline pH, EcoRI was found to cleave DNA at additional sites, typically N/AATTN (70). This ‘star activity’ (EcoRI*) was also observed in the presence of organic solvents, such as glycerol or DMSO (71–73), and when Mg^2+^ is replaced by Mn^2+^ (74). Co^2+^ and Zn^2+^ also support DNA cleavage, but unlike Mn^2+^ do not result in star activity (75). Preferred EcoRI* sites were identified to be GGATTT, AAATTT, GAATTT and GAATTA, whereas CAATTG resists attack (73). Later, Rosenberg and Greene (76) suggested that the hydrolysis rates of EcoRI* sites can be summarized by the hierarchies: G>>A>T>>C at the first position, and A>>[G,C]>>T at the second and third positions (and the corresponding complements at positions four, five and six). This was later quantitatively analyzed with synthetic oligonucleotides (77). Star activity turns out to be a general phenomenon, observed with other REases (e.g. 72,78–83).

Star activity is often also observed at high enzyme concentrations under optimum buffer conditions, and this reflects the finite accuracy of these enzymes. By analyzing the rate of cleavage of star sites on a plasmid DNA by EcoRV it was possible to estimate the accuracy of a REase. The plasmid pAT153 contains 12 EcoRV* sites, each of which differs from the wild-type EcoRV sequence (GATATC) by one base pair. EcoRV showed a marked preference for one of these sites (GTTATC), which was cleaved (*k*_cat_/*K*_m_) six orders of magnitude more slowly than the cognate site (GATATC). Nicked intermediate accumulates in the course of this cleavage. *In vivo*, this would enable DNA ligase to repair the single-strand breaks that arise at star sites (84). From cleavage studies with oligonucleotides, it was concluded that double-strand cleavage of non-cognate substrates is at least five orders of magnitude slower than cleavage of the cognate substrate (85). While in the cognate substrate both strands of the DNA duplex are cleaved at the same rate, in non-cognate substrates one strand is cleaved faster than the other one. These studies showed that REases are among the most accurate enzymes known. This high accuracy is achieved by both preferential binding (ground state) and preferential catalysis (transition state). Cleavage at star sites by high concentrations of enzyme can be suppressed to some extent by spermidine (86), hydrostatic pressure (87) and, as shown recently, by mutations (88).

#### The structural basis of specificity of REases: characterization of the REase–DNA interface using modified substrates

Because Type II REases recognize their substrate sequences so accurately, they are attractive subjects for studying the mechanism of recognition. It was unclear at the beginning of these studies how recognition occurred, and it remains incompletely understood today. Initially, it was speculated that recognition of symmetric (‘palindromic’) sequences might depend on unusual structures such as open, partially single-stranded, sequences (38) or cruciforms (89). Although DNA is almost always distorted to some degree when bound by REases, these deformations are thermodynamically unstable, and aside from a few unusual occurrences in recently solved crystal structures (e.g. PacI (22), and the EcoRII/PspGI/Ecl18kI/SsoII family (90)), they play little role in sequence recognition.

A decade before the first REase-DNA co-crystal structure (EcoRI) was solved, it was realized that in the DNA double helix, each base pair offers a unique pattern of contacts in the major and minor grooves that might enable base-recognition by ‘direct readout’, and also, perhaps, through additional contacts to backbone phosphate groups by ‘indirect readout’ (i.e. the recognition of a DNA sequence through the sequence-dependent conformation of the DNA backbone). X-ray crystallography of double-stranded RNA molecules, in conjunction with a systematic analysis of possible amino acid–base contacts, suggested that proteins might discriminate base pairs by the positions and polarities of hydrogen bonds (H-bonds) (91). From an experimental point of view, DNA molecules containing modified bases can be used to identify features within recognition sequences, such as H-bond donors and acceptors, or thymine 5-methyl groups, that REases might use for recognition. Disruption of such interactions by nucleotide methylation is the universal way that cells protect their own DNA from REase cleavage, naturally. Methylation of the EcoRI recognition sequence by the M.EcoRI methyltransferase (MTase), for example, changes the sequence from GAATTC to GA**m6A**TTC (m6A = *N*6-methyladenine) and this ‘modification’ completely protects the sequence from cleavage by EcoRI (48,92).

Analysis of naturally modified DNA molecules allowed some of the features of GAATTC that are important to recognition by EcoRI to be discerned. Non-glucosylated bacteriophage T4 DNA is cleaved partially by EcoRI, indicating that 5-hydroxymethylcytosine (5hmC) can be accepted instead of cytosine in GAATT**C** (93–95). Substituting hydroxymethyluracil (hmdU) for thymine lowers the maximal velocity of cleavage (*V*_max_) somewhat, but does not affect *K*_m_; substituting uracil (dU) instead affects neither *V*_max_ nor *K*_m_ (96). These results suggest that the 5-methyl groups of thymine are not *major* determinants for recognition by EcoRI. Substituting inosine for guanine likewise suggested that the minor groove 2-amino group of dG also does not play an important role in recognition by EcoRI in contrast to what was found for M.EcoRI (97). This implies that the recognition mechanism of the REase and its companion MTase differs, a situation now known to be true for all such pairs since they display little amino acid sequence similarity and frequently bind in different oligomeric forms, the one as a homodimer, for example, and the other as a monomer.

Synthetic oligodeoxyribonucleotides (oligos) became available In the early nineteen-seventies; solid phase synthesis was introduced somewhat later (98). The first cleavage experiment with EcoRI and synthetic oligos was performed with the self-complementary 8-mer pTGAATTCA, which was accepted as a substrate by both R.EcoRI and M.EcoRI (66). Oligos were subsequently used extensively to study structure–function relationships in the recognition process of EcoRI and other REases (77,99–110). Using oligos with modified bases, recognition of the same sequence by different enzymes could be analyzed and compared. For example, the thymine residues (probed by dU, hmdU and BrdU) in the EcoRI recognition sequence (GAA**TT**C) appear not to be directly involved in the recognition process by R.EcoRI, whereas they are important for M.EcoRI (96), and they are major points of contact for R.EcoRV (101). Similarly, it was shown that the isoschizomers HaeIII, BspRI and BsuRI, which recognize and cleave the same sequence, GG|CC, do so in different ways. Substituting dI for dG, and dU for dC, within the recognition site affected the rates of cleavage differently for all three enzymes (111).

Modified oligos were also important in analyzing the mechanistic and stereochemical aspects of catalysis by EcoRI (112–114) and EcoRV (115,116). In the words of a much respected pioneer in this field, through such experiments it was ‘*possible to discern the topography of the active sites of enzymes by examining substrate analogs for their ability to serve as reactants. Such investigations aim to contribute to our understanding of the kinetic and chemical mechanisms as well as the stereochemistry and stereoselectivity of a reaction*’ (117).

In a complementary approach, alkylation-protection, ethylation-interference, chemical-crosslinking, and UV- and chemical-footprinting experiments were carried out to probe the EcoRI–DNA interface (65,109,118–119). They showed that EcoRI protected the major groove N7 atom of dG, and the minor groove N3 atom of both dA residues within the EcoRI sequence against methylation by dimethyl sulfate. Ethylation-interference experiments showed that all but one of the phosphates within the recognition sequence, when alkylated, interfered with complex formation, and that two additional phosphates on each side of the recognition sequence also contacted the enzyme. The base and phosphate contacts were found to be symmetrically distributed about the dyad axis of the EcoRI sequence, demonstrating that the EcoRI dimer interacts with both strands of the EcoRI sequence equally.

### Biochemical characterization of REases

**Figure 3.**
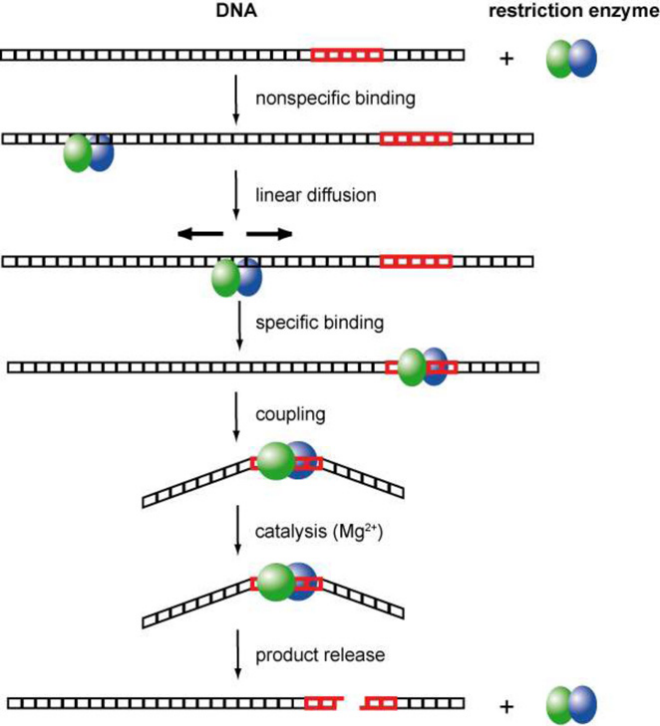
Schematic illustration of the steps involved in DNA recognition and cleavage by REases (120).

The catalytic reaction of a REase entails the following processes (Figure 3): (i) attaching to DNA non-specifically; (ii) locating the target sequence; (iii) recognizing and binding that sequence; (iv) coupling of recognition and catalysis; (v) cleavage of the sequence; and (vi) product release.

#### Steady-state kinetics

The first REase purified to homogeneity and rigorously characterized was EcoRI (121), which recognizes G|AATTC in double-stranded DNA and cleaves in the presence of Mg^2+^ ions at the position (‘|’) indicated (48,53). Its subunit molecular weight was determined to be around 30 kDa (122). In solution it exists in dimer–tetramer equilibrium with a *K*_d_ of 0.1 μM. Its Michaelis-Menten parameters toward ColE1 DNA at 37°C were found to be *k*_cat_ = 4 min^−1^ and *K*_m_ = 8 nM (121). In single-turnover experiments at high EcoRI concentrations, the catalytic constant for cleavage of each strand had the same value of 0.35 s^−1^ at 21°C (123). These data suggest that product release is rate limiting for EcoRI cleavage of macromolecular DNA substrates. The reason for this may be that the preferred way of dissociation of enzyme and product involves outside sequences (see below). Similar biochemical properties were described later for other Type II REases, particularly those of the Type IIP subclass (124–126), although in some cases, most notably for Type IIT REases (127,128), the two strands are not cleaved simultaneously, and nicked intermediate can accumulate (126,129). Yet other Type IIP REases are monomers that cleave the two DNA strands sequentially, one after the other, in separate catalytic events (130–133).

#### Thermodynamics and kinetics of DNA binding

The affinity of a REase for its substrate sequence was determined for EcoRI using the nitrocellulose filter-binding technique that had been developed in the mid-1960s (134,135). Experiments with EcoRI, and with other REases, were carried out in the absence of Mg^2+^ to prevent cleavage (see (136–139) for early reviews). At 37°C, affinity to pBR322 (with one EcoRI site) decreases with increasing ionic strength: at 0.07–0.15 M, *K*_d_ lies between 10^−11^ and 10^−10^ M (139). With λ DNA (with one EcoRI site) a *K*_d_ of 10^−9^ M was determined at 22°C and an ionic strength of 150 mM (138). The parameter measured in these experiments is an apparent *K*_d_, as it does not take into consideration that non-specific DNA binding accompanies specific binding. Using a protection assay, the *K*_d_ for non-specific binding of EcoRI to ΦX174 DNA (with no EcoRI sites) was determined to be in the range of 10^−6^ M (nucleotides) at an ionic strength of 200 mM and at 20°C (140). Non-specific binding was also analyzed by a competition-cleavage assay with synthetic polynucleotides in the presence of Mg^2^^+^ and the *K*_d_ was found to be 10^−4^–10^−5^ M (nucleotides) (141). Strong *specific* binding in the nM to pM range, and relatively weak *non-specific* binding in the μM range, was found to be true of REases in general. While EcoRI and most other Type II REases bind to their recognition sequence specifically even in the absence of Mg^2+^, EcoRV binds all DNA sequences with equal affinity in the absence of Mg^2+^ (142). As was shown by the newly developed gel electrophoretic mobility shift assay (143,144), Mg^2+^ and other divalent metal ions, particularly Ca^2+^, confer specific binding ability on EcoRV (145). Today, this assay (‘EMSA’) has largely replaced the nitrocellulose filter binding technique for analyzing the binding of proteins to nucleic acids.

Formation of the non-specific complex and transition to the specific complex is accompanied by changes in solvation and counter-ion binding. For EcoRI, the non-specific complex was found to sequester around 110 more water molecules than does the specific complex with the recognition sequence (146). This indicates that the association between the protein and the DNA is much tighter in the specific complex than in the non-specific complex, with only a small number of water molecules present at the protein–DNA interface.

#### Facilitated diffusion, linear diffusion, sliding and hopping

Detailed investigation of the kinetics of the EcoRI-substrate interaction revealed a surprising result (10,147). Whereas the affinity, *K*_d_, of EcoRI to pBR322, a 34 bp oligo derived from pBR322 containing one EcoRI recognition site, and the double-stranded dodecamer p(CGCGAATTCGCG) varied between 5 × 10^−12^ and 15 × 10^−12^ M, the dissociation rate constants, *k*_d_, for complexes of EcoRI and DNA were much more dependent on the chain length of the DNA (148). This led Modrich *et al.* to conclude that outside DNA sequences are involved in the major kinetic path by which EcoRI locates and leaves its recognition sequence (148). This was interpreted in terms of facilitated diffusion (149,150), meaning that EcoRI locates its recognition sequence by first binding to DNA non-specifically, and then sliding along the DNA randomly until it encounters the sequence. Likewise, EcoRI leaves its recognition site, to which it binds firmly, via non-specific sliding. Facilitated diffusion is also observed in the presence of Mg^2+^, as shown by analyzing the DNA cleavage-rate dependence for substrates of different length (148). It was shown that the mean diffusion length of EcoRI is approximately 1000 bp at 1 mM MgCl_2_; similar results were obtained for HindIII and BamHI (151), and later confirmed for BamHI (152), and demonstrated by different techniques for EcoRV (153,154) and BssHII (155).

Linear diffusion is critically dependent on contacts between amino acid side chains of the protein and the backbone of the DNA. Changing the centro-symmetric electrostatic potential in the DNA-binding site affects sliding. It was demonstrated that the presence of other proteins bound to the DNA, and of irregular DNA structures such as bent DNA or a triple helix, constitute a barrier that cannot easily be passed by EcoRI (151,154). Although DNA in the cell is packed with other proteins, facilitated diffusion is still essential for *in vivo* function, as shown for EcoRV by correlating the phage restriction activity and the linear diffusion rate of EcoRV variants (156). Sliding of REases is likely to follow the pitch of the double helix. This was experimentally verified for EcoRV. The enzyme tends to overlook cleavage sites at 1 mM MgCl_2_ (which could be the consequence of hopping) but not at 10 mM MgCl_2_, which indicates that under these conditions sliding predominates (153).

The mechanisms of facilitated diffusion have been of continuous interest to the present day. As pointed out by Modrich *et al.* (147), facilitated diffusion of REases could involve one-dimensional sliding as well as hopping, as originally proposed (149). For some REases it has been argued that the principal mode of transfer is by ‘hopping’ and ‘jumping’, i.e. the dissociation of the protein from one site followed by its re-association with another site in the same DNA molecule, either close to or distant from the original site (157). There are a variety of ways to analyze facilitated diffusion of REases and its contribution to target location (158,159). Single-molecule experiments are particularly useful for this purpose and substantiate that sliding alternates with hopping/jumping during facilitated diffusion of EcoRV (160,161). The extent to which REases make use of one-dimensional or three-dimensional diffusion for target site location depends on the ionic strength and the Mg^2+^ concentration (153). The actual path length for sliding, and the effect of salt on this process, are likely to vary from protein to protein (162).

### Cloning and sequencing of the genes coding for REases

Five years after EcoRI was purified to homogeneity in 1976, the amino acid sequences of the EcoRI REase and MTase were determined by cloning the EcoRI R–M system and sequencing its two genes (163,164). R.EcoRI was found to comprise 2 × 277 amino acids (subunit molecular mass, *M_r_* = 31,063 Da), and M.EcoRI to comprise 1 × 326 aa (*M_r_* = 38,048 Da). 31 kDa is a typical subunit size for a Type IIP REase, which ranges in size from PvuII (recognition sequence: CAG|CTG; subunit *M_r_* = 18.3 kDa), on the small side, to ClaI (AT|CGAT; subunit *M_r_* = 41.6 kDa), on the large side. No aa sequence similarity was found between the EcoRI REase and MTase, even though they recognize the same DNA sequence, suggesting that the two proteins had different evolutionary origins (164). Lack of similarity between REases and their companion modification enzymes has since been found to be true for all R–M systems of this kind, suggesting that R–M systems arose by gene associations rather than by gene duplications and divergence.

Following the cloning of EcoRI, the genes of many more Type II REases were cloned, sequenced and compared. Cloning brought many benefits. Genes could be moved from poorly characterized organisms to more convenient hosts such as *E. coli* K12. They could be sequenced, studied and altered. Their proteins could be separated from contaminating enzymes present in the original host. And, by increasing gene copy number and expression rates, they could be produced in greater quantities. Molecular biologists were quick to apply gene cloning to the very enzymes that made cloning possible, including DNA ligases (165–168), DNA polymerases (169,170) and restriction enzymes; see (171,172) for early reviews. Almost all of the enzymes available commercially today for DNA manipulation and analysis—including over 250 REases—are purified from overexpression clones. As a result, these reagents are much purer and less expensive than they were, and in the process a great deal has been learned about their biology, genetics and biochemistry. Perhaps no other class of enzymes has been investigated as extensively as Type II REases.

Cloning REases presents several challenges. Foremost is their toxicity. Cells protect themselves from restriction by methylating each recognition site in their own DNA. This ‘modification’ is catalyzed by the MTase(s) that partner with restriction enzymes *in vivo* to form R–M systems. In order to clone an REase, its partner MTase(s)—there can be more than one—must also be cloned to prevent destruction of the new host's DNA. Fortunately, perhaps due to eons of natural selection for efficient lateral gene transfer between prokaryotes, the genes for the REase and its accompanying MTase(s) are usually closely linked. This allowed many R–M systems to be cloned in one step, on DNA fragments that contained both genes. Among these were HhaII (173,174), EcoRII (175,176), EcoRI (164,177), PstI (178,179), PaeR7I (180–182), EcoRV (183,184), PvuII (185) and BsuRI (186). Some of these systems occurred on plasmids and were isolated by simple sub-cloning. Others were chromosomal, and were isolated by selecting for phage-resistance, for insensitivity to restriction (187) or for resistance to REase-digestion (188). See (189) for a brief discussion of early cloning methods.

When R–M systems are cloned, the recipient cell can be exposed to the new REase before its DNA becomes fully modified. Cells can cope with this in some cases (18), but in others they cannot, and when this occurs the system must be cloned in two steps. The MTase gene must be cloned first, and the cells allowed to become fully modified before the REase gene is introduced on a separate vector. DdeI (190), BamHI (191) and BglII (192) were early examples of this situation. In addition to genes encoding the REase and MTase(s), many R–M systems include a gene for a ‘controller’ protein. These C-proteins are transcriptional regulators that are thought to coordinate gene expression during natural lateral transfers to avoid premature REase synthesis (193–196).

Another challenge to cloning R–M systems concerns the MTases themselves. Some strains of *E. coli* cannot tolerate certain kinds of DNA methylation. MTases that catalyze such modifications, and the R–M systems to which they belong, cannot be transformed into these hosts, whereas they can into other strains such as HB101 and its derivative, RR1 (197–199). This intolerance was traced to two endogenous *E. coli* systems, termed RglA and RglB, first encountered in connection with the restriction of non-glucosylated bacteriophage T4 (200). The DNA of this phage contains 5hmC instead of cytosine, and the Rgl systems were thought to attack 5hmC-containing DNA, exclusively. In fact, it was found, they also attack DNA containing 5-methylcytosine (5mC) in certain sequence contexts, and since 5mC-modification is catalyzed by many R–M systems, these systems are incompatible with Rgl-proficient cells.

The Rgl systems were renamed McrA (modified cytosine restriction) and McrB (later McrBC) to more accurately reflect their specificities (198,201). McrA restricts modified DNA in the context of the HpaII recognition sequence, C5(h)mCGG. It is a small HNH-type endonuclease (202–204)), but has not been well characterized. McrBC restricts modified DNA in the context R5(h)mC (R = A or G) and is well characterized. These enzymes are examples of a growing collection of ‘modification-dependent’ REases, now termed ‘Type IV’, that includes Mrr (205–207), MspJI (208), PvuRts1I (209–212), GmrSD (213) and BisI (214), which we are learning are ubiquitous in bacteria. See (3) for a recent review.

Scientific progress depends on insight and careful experimentation and also sometimes, as Mcr exemplifies, on plain good luck (43). HB101/RR1 and K802 were popular *E. coli* cloning hosts at the time and were used for most of the early R–M cloning experiments. As was eventually discovered, HB101/RR1 is defective in McrBC and Mrr, and K802 is defective in McrBC and McrA (206). The fortuitous choice of these hosts allowed many R–M systems to be cloned, and thence the existence of the Mcr systems to be discovered. Had alternative popular cloning hosts of the time been used instead, such as MM294 (McrA^+^, McrBC^+^, Mrr^+^), attempts to clone R–M systems would frequently have met with failure, and this would have set the effort back considerably.

Several procedures were used to clone Type II R–M systems. The customary starting point was a plasmid library containing partial-digestion fragments of total bacterial or archaeal DNA (Supplementary Figure S1). The libraries were grown to allow plasmids carrying MTase genes to modify themselves. The plasmid pools were purified, and then digested *in vitro* with the REase whose gene was to be cloned in order to destroy unmodified plasmids, but leave modified plasmids intact. The digests were re-transformed, and survivors were screened individually, or pooled and cycled through another round of selective REase-digestion. This procedure, termed ‘methylase-selection’ or, whimsically, ‘the Hungarian trick’ (189), is a more general version of the method used to clone the first MTase, M.EcoKI (187). It was suggested by Mann *et al.* as a possible alternative to the phage-resistance method that they used to clone HhaII (174). The procedure reliably yields MTase genes, it was found (188,215–217), but often not complete R–M systems.

Libraries were also exposed to phages to select for cells able to restrict because they carried complete R–M systems (178). This ‘phage-selection’ method frequently failed, however, likely due to inadequate R-gene expression (218). When methylase-selection yielded only the M gene, adjacent overlapping fragments were identified by Southern blots, mapping, inverse PCR and sequencing, in order to obtain the R gene. N-terminal amino acid analysis of purified REases, and internal tryptic peptide analysis, were often used to identify the correct open reading frame. Between 1980 and 2005, several hundred Type II R–M systems were cloned and analyzed, some in academic laboratories, but most in the research laboratories at New England Biolabs (NEB) in the United States, and at Fermentas (now part of Thermo Fisher Scientific) in Lithuania. Since then, with the advent of inexpensive genome sequencing using 454 Life Sciences machines (Roche), and more recently PacBio single-molecule real-time (SMRT®) machines (Pacific Biosciences), many R–M systems have been cloned by identifying their genes through bioinformatics analysis of whole-genome sequences, and then retrieving them by PCR or by gene synthesis. PacBio offers an advantage in this regard because it not only generates the DNA sequences of the R–M systems present but also, through methylome analysis, often the recognition sequences of those same systems (219).

As information about the organizations, genes and proteins of R–M systems accumulated as a result of cloning, an online dedicated database was created by Rich Roberts and Dana Macelis with funding from the National Library of Medicine (220). REBASE has been continuously improved over the years and is updated almost daily with new data on R–M systems of all types including putative systems identified in genomic sequences by bioinformatics analysis, and very recently, with PacBio methylome information. Despite its folksy homepage, REBASE (http://rebase.neb.com/rebase/rebase.html) is an encyclopedic source of expert knowledge on all things related to restriction and modification (7). Most of the R–M systems that have been cloned and characterized have not been formally published. Their sequences are nevertheless available in REBASE, for the most part, and when they are not, they can be provided upon request. A list of the R–M systems cloned by various groups at NEB is given in Supplementary Table S1.

### Evolution of Type II REases

Except for isoschizomers, Type II REases were found to share surprisingly little aa sequence similarity. This led many researchers to believe that, for the most part, they are *not* evolutionarily related. One of the earliest examples of clear aa sequence similarity between REases was found between EcoRI and RsrI, which catalyze the same reaction: G|AATTC (221). The aa sequences of these two enzymes are identical in several places and 50% identical overall (222). It was perhaps not surprising, then, that catalytically active hybrids of these two isoschizomers could be formed (223). A common evolutionary origin seems indisputable for these two enzymes, as it also does for other pairs of isoschizomers such as MthTI, NgoPII and FnuDI (224), and XmaI and Cfr9I (171,225). A systematic statistical analysis of the phenotype (substrate composition, length and cleavage position) of REases on one hand and the genotype (amino acid sequence) on the other (226) suggested that REases of the PD….D/EXK family are frequently the products of divergent evolution. Furthermore, comparison of codon usage among REases and their companion MTases (227) indicated that horizontal gene transfer has contributed to the wide distribution and evolution of Type II R–M systems in general. Ichizo Kobayashi and colleagues at Tokyo University have shown that R–M systems can act as selfish genetic elements and that this might have contributed to the evolution of R–M gene pairs (228). The notion that apparently disparate REases might nevertheless be evolutionarily related, in some instances, grew more compelling when crystal structures of REases became available and revealed that the catalytic site for DNA cleavage (‘the common core’) was structurally similar in many of them (229–231). Multiple alignments of REase aa sequences sometimes shows sequence similarities over short stretches of a few amino acids, likewise suggestive of perhaps common, if distant, evolutionary origin (232–234).

BfiI (235) was the first REase found that did not belong to the PD…D/EXK catalytic family; it belongs to the phospholipase D superfamily (236) instead and, unique among REases, does not require a divalent metal ion such as Mg^2+^ for cleavage. There is clear evidence from bioinformatics and structural studies that several other Type II REases do not belong to the PD…D/EXK family, either. KpnI (GGTAC|C) (237), Hpy99I (|CGWCG) (238) and PacI (TTAAT|TAA) (22) belong to the ‘HNH’-endonuclease family that includes Holliday junction resolvases. (These are also referred to as ‘beta beta alpha-metal fold’ REases due to the presence of Cys4 Zn^2+^-binding structural elements.) Eco29kI and Cfr42I (CCGC|GG) (21,239), and Hpy188I (TCN|GA) (240,241) belong to the ‘GIY-YIG’-family that also includes many homing endonucleases (233,242). We discuss these catalytic classes briefly later. Type II REases are currently grouped into several subtypes. These subtypes do not necessarily represent separate branches on the REase evolutionary tree. For example, SsoII (Type IIP; |CCNGG), EcoRII (Type IIE; |CCWGG) and NgoMIV (Type IIF; G|CCGGC) have remarkably similar DNA-binding sites and catalytic centers (234). Specificities for partly related, and even unrelated, sequences can nevertheless depend upon the same structural framework: CCNGG (SsoII), CCWGG (PspGI/EcoRII), GCCGGC (NgoMIV), RCCGGY (Cfr10I), GATC (MboI) (243).

### Large-scale purification of REases from overproducing *E. coli* strains

Overproduction of EcoRI, EcoRV and other REases was of great importance for the biochemical study of these enzymes. EcoRI, for example, could be isolated in gram quantities from an overproducing strain rather than milligram quantities from the wild-type bacterium (244). In some constructs (245), overproduction of EcoRI resulted in inclusion body formation. EcoRV overproduction yielded a soluble protein preparation (183). Introduction of polyhistidine-tags at the N-terminus or C-terminus of recombinant REases enabled rapid, small-scale partial-purifications by metal chelate chromatography (246) and increased the speed with which REases and their engineered derivatives could be isolated and purified manyfold. Overproduction was, in many cases, the prerequisite for a crystallographic analysis.

### Crystal structures of REases in complex with DNA

**Figure 4.**
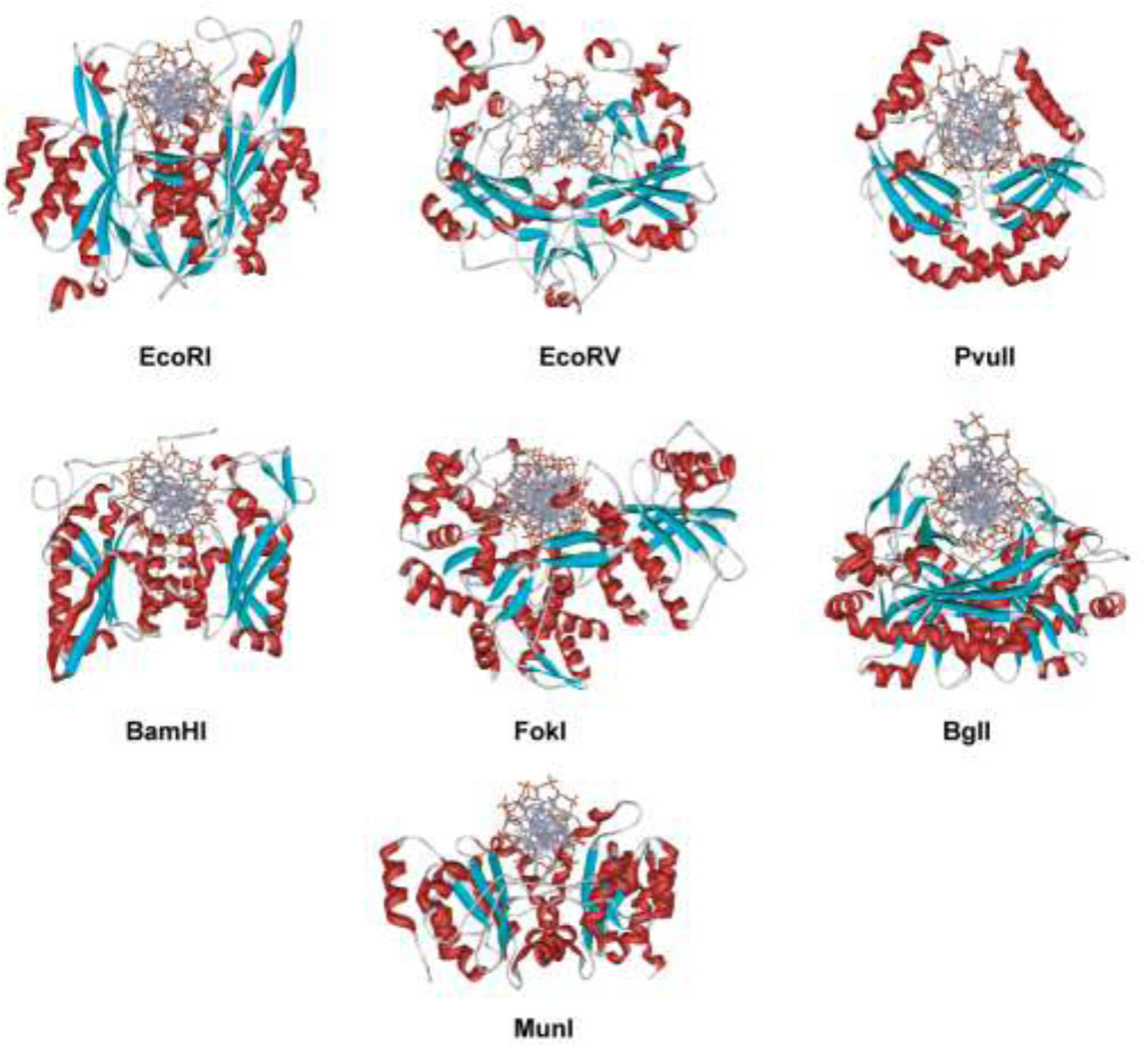
Co-crystal structures of specific restriction enzyme–DNA complexes determined between 1990 and 1999.

The first REase crystal structure, that of EcoRI, was reported in 1986 (247). The enzyme was crystallized with self-complementary 12- and 13-mer oligos in the absence of Mg^2+^ to avoid DNA cleavage. Although the 3 Å resolution of the structure was low by today's standards, it represented the first detailed picture of a protein interacting with its recognition sequence at the atomic level. This structure generated intense interest (247) and immediately sparked site-directed mutagenesis experiments aimed at studying these interactions. The methodology of site-directed mutagenesis had been developed by Smith *et al.* a few years earlier (248). Mutational analysis was carried out both to verify the proposed recognition and cleavage mechanisms, and to rationally alter the sequence specificity of EcoRI, if possible, by changing the amino acids that form its binding site (249–257). The results of these experiments contradicted some aspects of the structure, prompting this to be re-examined, and subsequently revised (258). Over the next decade, the co-crystal structures of six more Type II REases bound to their recognition sequences were solved to increasingly higher resolution. These included EcoRV (259), PvuII (260), BamHI (261), FokI (262), BglI (263) and MunI (264) (Figure 4). Over 30 REase-DNA co-crystal structures have now been solved and represent a substantial, if underused, collection of material for further study (Supplementary Table S2).

Comparison of the EcoRI and EcoRV co-crystal structures, and structure-guided site-directed mutagenesis, showed that the two enzymes had similar PD-(D/E)XK active sites (265,266), and similar overall folds comprising β-sheets sandwiched between α-helices (231). This fold, a central, four-stranded mixed β-sheet flanked by two α-helices on both sides (with αβββαβ topology), was subsequently found, with variations, (267,268) in almost all Type II REases whose structures have been determined. This fold is classified in the SCOP (Structural Classification of Proteins) database [http://scop.mrc-lmb.cam.ac.uk/scop] as the REase-like fold. Recent bioinformatics analysis (26) indicated that among 289 experimentally characterized Type II REases, whose full-length sequences were available, 69% belonged to the PD-D/EXK phosphodiesterase superfamily that includes other nucleases such as λ-exonuclease, RecB endonuclease, *Sulfolobus solfataricus* Holliday junction resolvase, MutH, T7 endonuclease I, and VSR endonuclease.

### The recognition process as deduced from co-crystal structures

The crystal structures of specific complexes formed between REases and oligos containing their recognition sequence are presumed to be representative of the recognition event, even though the essential metal cofactor Mg^2+^ is usually absent or substituted by the catalytically inactive Ca^2+^ or Na^+^. In most structures, the bound DNA is distorted to some degree from B-form DNA, and in some—MspI, for example (269), and PacI (22)—changes seem to have occurred during crystallization that obscure the recognition event. Nevertheless, REase co-crystal structures are the basis for our efforts to understand the recognition process. It should be kept in mind that *at best* these give only a snapshot of what is a dynamic process, and only an idea of what the transition state looks like. The recognition process begins with complex formation, and ends with the catalytic action.

#### EcoRI

**Figure 5.**
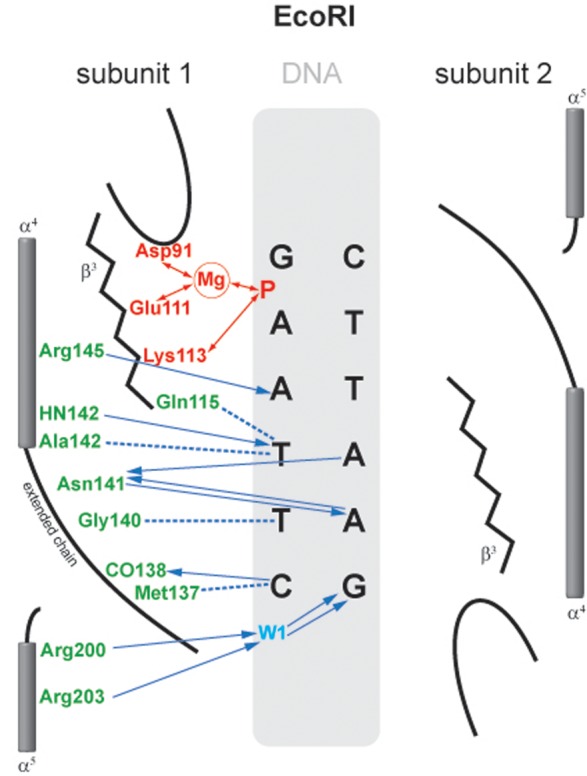
Schematic representation of the interaction of EcoRI with its recognition sequence. For clarity, interactions with only one subunit are shown; those with the other subunit are identical and symmetric. Hydrogen bonds and polar interactions are shown as arrows, van der Waals interactions as dotted lines. Amino acids and interactions involved in catalysis are depicted in red; those involved with sequence-recognition are depicted in green and blue (120).

Upon specific complex formation with EcoRI, the DNA becomes kinked and unwound within the AATT sequence. The two central base pairs of GA**AT**TC are unstacked and wedged 55° apart by insertion from the major groove of the Ala 142 side chain methyl group from each subunit, which also widens the major groove. Overall, the DNA is bent by about 12°. Facilitated distortion of the DNA site enhances EcoRI–DNA recognition, a subtlety of the recognition mechanism true for many other REases (114). The central distortion of EcoRI, for example, nudges the adjacent AT and TA base pairs there into better alignment with the side chain of Arg 145, and the main chain atoms of Asn 141 and Ala 142 with which they form H-bonds. Several structural elements of EcoRI are involved in DNA contacts (Figure 5): (i) a bundle of four α-helices, two from each subunit, penetrate the widened major groove and make base and backbone contacts at their amino termini; (ii) an extended chain runs through the major groove of the recognition site; (iii) a β-strand running parallel to the DNA backbone contains amino acid residues essential for catalysis and amino acid residues engaged in phosphate contacts; (iv) two arms reach around the DNA and are responsible for backbone contacts outside of the recognition sequence. These contacts outside of the recognition sequence may explain why EcoRI cleaves its sites on DNA with different rates depending on the adjacent sequences (58–59,62–63).

Altogether, there are 16 protein-base H-bonds (12 to purines and 4 to pyrimidines), and 6 van der Waal's contacts (to the pyrimidines), all in the major DNA groove. In addition to these base-specific contacts (‘direct readout’), there are numerous contacts to the backbone of the DNA that could recognize the specific sequence through sequence-dependent backbone conformation (‘indirect readout’) (270). These contacts play a very important role in coupling recognition to catalysis and in coordinating the two catalytic sites (271). Thus, the recognition process is redundant, with multiple direct and/or indirect contacts to each base pair. Many of these contacts were probed by site-directed mutagenesis experiments, which have confirmed their importance for the recognition process (249,254–256,272–277). In general, mutation of amino acids involved in base-specific contact results in a large reduction in activity, but not to a change in specificity. That these contacts can be removed without reducing the accuracy of discrimination indicates that the recognition process is highly redundant, and might also depend upon steric exclusion and structural factors of the kind referred to as ‘appositional interactions’ (278,279). It must be emphasized that a mutational analysis of the protein–DNA contacts is at best qualitative because amino acid substitutions inevitably perturb the protein structure, and likely also alter the arrangement of water molecules at the protein–DNA interface. Specific complex formation was analyzed by fast kinetics. EcoRI and the substrate were found to associate in the presence of Mg^2+^ in a nearly diffusion-controlled process (280).

#### EcoRV

The structure of EcoRV, the next to be crystallized after EcoRI, was solved in multiple forms, including the free enzyme (apo-protein), specific enzyme–DNA complexes, an enzyme–product complex and, revealingly, a non-specific complex (259,281). BamHI is the only other REase for which such a range of structures is available (261,282). Comparison of the non-specific and the specific EcoRV complexes reveals the conformational changes that accompany recognition. EcoRV induces a striking distortion from regular B-form DNA. The resulting strained conformation is characterized by a ∼50° central kink, unwinding of the DNA, unstacking and twisting of the central two base pairs of GA**TA**TC by intrusion into the minor groove of the Lys 38 side chain from each subunit, and bending of the DNA making the major groove narrow and deep and the minor groove wide and shallow. The EcoRV-induced bending of specific DNA had been confirmed by gel shift assays with an inactive EcoRV mutant in the presence of Mg^2+^ (283), the wild-type enzyme in the presence of Ca^2+^ (284), and by scanning force microscopy (285).

**Figure 6.**
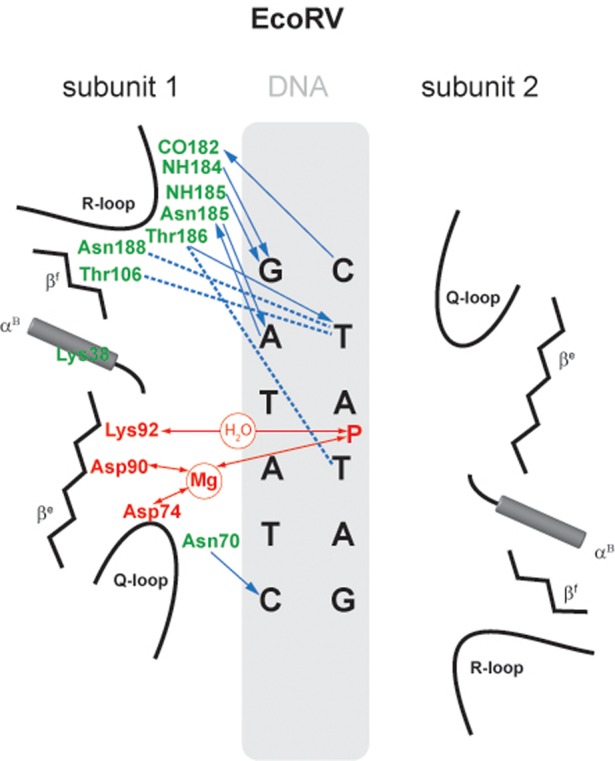
Schematic representation of the interaction of EcoRV with its recognition sequence. Interactions with only one subunit are shown; those with the other subunit are identical and symmetric. Amino acids and interactions involved in catalysis are depicted in red; those involved with sequence-recognition are depicted in green and blue (120).

The conformation of the EcoRV protein itself also changes during transition from the non-specific to the specific complex, a feature we now know to be common among REases. These changes include reorientation of two subdomains allowing EcoRV to encircle the DNA, and ordering of three loops that are disordered in the free protein and the non-specific complex, two of which are involved in recognition by making specific contacts to the DNA in the major and minor grooves. The principal recognition elements of EcoRV, the R-loops, engage in 12 out of 18 possible major groove H-bonds with the bases, two van-der-Waal's contacts to the methyl group of the outer thymidines (GATA**T**C) and 12 wateR–Mediated H-bonds to the DNA backbone (these numbers refer to both subunits and double-stranded DNA). The other important recognition element, the Q-loop, forms two H-bonds to the bases in the minor groove and harbors the catalytically important residue Asp74 (Figure 6).

It is noteworthy that in the specific EcoRV–DNA complex, no H-bond interactions are present in the major groove with the two central base pairs (GA**TA**TC). Compression of the major groove at this position due to the 50° kink limits direct access. Numerous contacts occur between the protein and the DNA backbone. Not including the R- and Q-loops, approximately 24 amino acid side chains with H-bond donor capacity or positive charge are sufficiently close to phosphate groups to interact favorably. Some of these contacts are to phosphates outside of the recognition sequence, and might be responsible for the flanking sequence preferences of EcoRV (84,286). The mechanism of DNA recognition by EcoRV inferred from the crystal structure has been extensively investigated by site-directed mutagenesis (77,257,266,286–289). This has shown that substitution of amino acids involved in base-specific contacts results in almost inactive variants. Using chemically modified oligos (101,290–295), and oligos with degenerate recognition sequences (85), the importance of all of the exocyclic groups in the major groove of the recognition sequence has been examined. The inner AT base pairs (GA**TA**TC), which do not have direct contacts with the enzyme, were found to be as important for the recognition process as the other base pairs (**GA**TA**TC**). This implies that H-bond, and van der Waals, interactions with the protein are not the only way sequence recognition can occur, and that additional factors, such as conformation-dependent contacts to the DNA backbone (‘indirect readout’), and steric exclusion, can also be determinants. It is plausible that the propensity of the EcoRV recognition sequence to adopt an extreme bend between the central base pairs could exclude other DNA sequences from productively interacting with this enzyme (259). GC or CG base pairs are thought unlikely to allow such an extreme deformation as AT and TA base pairs. The role of phosphate contacts for the specific interactions of EcoRV and its target sequence was systematically analyzed by site-directed-mutagenesis experiments (286). The complete catalytic cycle of EcoRV has been observed by fast kinetics. EcoRV and its substrate associate in the presence of Mg^2+^ in a nearly diffusion-controlled process, and the binding and bending steps occur at equivalent rates (296). Positively charged C-terminal subdomains of EcoRV contribute to DNA binding, bending and cleavage (297). Binding may occur in two steps: non-specific binding to the C-terminal subdomain, followed by opening of the binding cleft and specific binding (298).

Common features of the EcoRI and EcoRV co-crystal structures allowed certain generalizations to be made concerning Type II REases, and their interactions with recognition sequences. These were soon confirmed, and extended, by the co-crystal structures of PvuII and BamHI, and then by others that followed.

1. The structures possess 2-fold rotational symmetry, as suggested by Hamilton Smith in his Nobel Lecture (299). This agreed with experimental results showing that protein contacts to the two half-sites of the palindromic recognition sequence were symmetric (65) and that the two identical subunits of EcoRI cooperate in binding and cleavage (52,300).
2. The substrate DNA is bound in a high energy conformation with large deviations from a B-form DNA. The DNA is kinked, though overall straight in EcoRI, and bent in EcoRV. The DNA is underwound and the base pairs are partially unstacked. Distortion is part of the recognition process, and is accompanied by conformational changes of the protein (296,301–304).
3. The protein–DNA interface is characterized by an intricate set of interactions with both bases and the phosphates. Most of the H-bond donor or acceptor atoms in the major groove of the recognition sequence are involved in H-bonds to the protein, some of them wateR–Mediated (see also (146)). In addition to interactions with bases, there are numerous interactions with the backbone, within and just outside the recognition sequence (286,305). Secondary, or buttressing, interactions support primary ones by properly positioning the amino acids that contact the bases or the backbone.
4. Primary and secondary interactions form an extensive network likely established in a highly cooperative manner during the recognition process.
5. The recognition process is redundant in that contacts to the base pairs are over-determined. Redundancy ensures that recognition is reliable, and implies that attempts to alter specificity by changing individual contact amino acids are unlikely to succeed, as has been amply demonstrated (306,307).
6. The catalytic site residues of EcoRI and EcoRV comprise two acidic amino acids and one lysine, located on the second and third β-strands: D91, E111 and K113 for EcoRI; D74, D90 and K92 for EcoRV.

There were also notable differences between the EcoRI and EcoRV structures.

1. EcoRI approaches the DNA, and likely tracks it, from the major groove. The minor groove is empty with no protein–DNA contacts. EcoRV approaches the DNA from the minor groove, and encircles it by wrapping arms into the major groove. These differences were later found to be typical for the α- (e.g. BamHI, BglII, Bse634I, BsoBI, Cfr10I, **EcoRI**, EcoRII, MunI, FokI, NgoMIV) and β- (e.g. BglI, **EcoRV**, HincII, MspI, NaeI, PvuII) evolutionary branches of the PD…D/ExK REases.
2. For EcoRI, contacts to the major DNA groove are made by an extended β-sheet and a ‘four barreled’ helix. For EcoRV, the major groove contacts originate from two loops.

Structures of non-cognate complexes of REases are available for only two REases: EcoRV (259) and BamHI (308). In both cases, the structure of the non-cognate complex is more open than that of the cognate complex. For BamHI, it was concluded that the structure of the ‘*non-cognate complex provides a snapshot of an enzyme poised for linear diffusion*’ (308).

### The mechanism of catalysis

**Figure 7.**
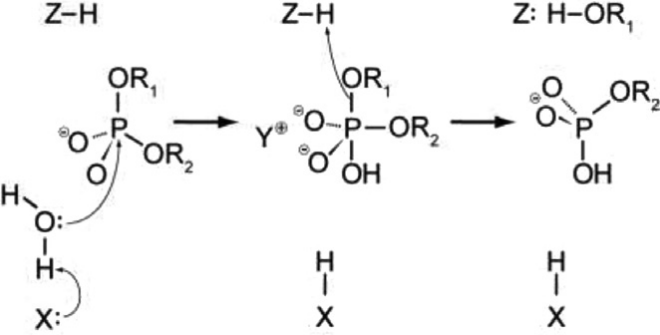
A general mechanism for DNA cleavage by EcoRI and EcoRV. An activated water molecule attacks the phosphorous in-line with the phosphodiester bond to be cleaved by an SN2 reaction, which proceeds with inversion of configuration. X, Y and Z are a general base, a Lewis acid and a general acid, respectively.

One of the most important questions regarding the catalytic mechanism of a hydrolase is whether hydrolysis involves a covalent intermediate, as is typical for proteases. This can be decided by analyzing the stereochemical course of the reaction. This was done first for EcoRI (112), and later for EcoRV (116). Both enzymes were found to cleave the phosphodiester bond with inversion of stereoconfiguration at the phosphorus, which argues against the formation of a covalent enzyme–DNA intermediate (Figure 7). Bfi is the only REase known to catalyze a transesterification reaction on DNA with retention of configuration at the phosphorus, which is indicative of a two-step mechanism. BfiI has a rare phospholipase-D catalytic site and has been shown to cleave the two DNA strands sequentially in a highly unusual manner that involves covalent enzyme–DNA intermediates (309).

**Figure 8.**
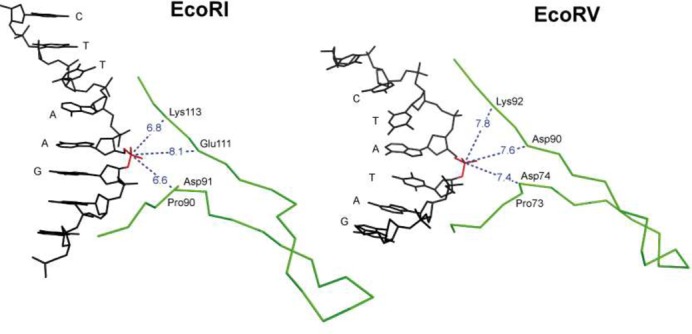
The active site (PD…D/ExK) of EcoRI and EcoRV.

Crystallographic analyses of the specific complexes of EcoRI (258) and EcoRV (259,281), in combination with mutagenesis, identified the catalytic sites of these enzymes (265). They were found to be closely similar in structure and behavior (Figure 8). Comparable catalytic sites were later found in other REases when their crystal structures were determined. These sites contained the signature ‘PD…D/EXK’ motif, a motif that occurs in many variations and can be difficult to identify in the absence of structural information because the two components, PD and D/EXK, are not invariant, and can be separated (‘…’) by anywhere from 4 (BcnI) to 51 (SgrAI) amino acids. Compounding matters, in some enzymes, the D/E or K residues, are recruited from other parts of the protein (e.g. EcoRII (310); BspD6I (311)). The importance of the acidic and basic amino acid residues for cleavage activity has been confirmed many times by site-directed mutagenesis (77,254–255,266,274,312), although their role is not fully established, and the precise mechanism of catalysis is still subject to interpretation. When crystallized with metal ions (Mg^2+^, Mn^2+^, Ca^2+^ or Na^+^), one ion is consistently found at the same position in the catalytic site, coordinated to one non-bridging oxygen atom (always proS) of the target phosphate, and up to five other oxygen atoms from the side chain carboxylates of the acidic residues, D and D/E; the main-chain carboxyl of residue X; and water molecules. The metal ion is thought to stabilize the transition state by neutralizing the build-up of negative charge on the phosphorus. Often a second ion is present, too, close to the 3′-leaving group, but its position varies somewhat. The lysine residue (K), which in some REases is replaced by E (e.g. BamHI (261)), Q (e.g. BglII (313) and NotI (25)), or even N (e.g. MspI (269)) might stabilize the transition state. Some also consider this to be the general base which de-protonates a water molecule to create the attacking hydroxide ion, although others argue that this is unlikely.

**Figure 9.**
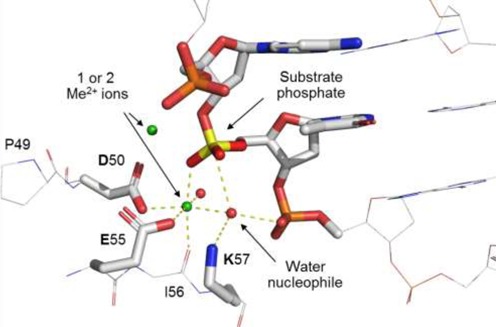
An example of a REase catalytic site (MvaI, pdb: 2OAA). The nucleophilic water is oriented with tetrahedral geometry to ‘attack’ the phosphorus: one H-bond is to K87 and one H-bond to the 3′-phosphate oxygen, both of which might act as the general base. One lone pair orbital of the attacking water is to the metal ion, and one lone pair orbital to the phosphorus atom.

**Figure 10.**
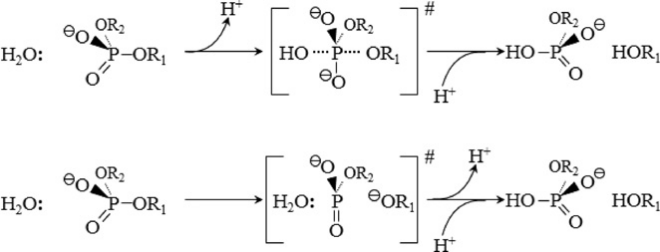
Alternative mechanisms of phosphoryl transfer reactions: associative (top) and dissociative (bottom). The mechanisms differ in the order of bond formation and breakage, and in the nature of the transition state (317).

In what is termed ‘substrate-assisted catalysis’ (314), the phosphate group 3′ to the hydrolyzed phosphate is another candidate for the general base in EcoRI and EcoRV (113), and also other REases (e.g. EcoO109I (315)). In EcoRV, two other carboxylates—not those of the PD…D/EXK motif—were discussed as being responsible for water activation (316). Alternatively, the attacking water could be activated by a water molecule from the hydration sphere of the Mg^2+^ ion at the catalytic center, or be one of a hydration sphere water molecules itself (e.g. MvaI, Figure 9 and BcnI (131,132)). Rosenberg *et al.*, who have been able to follow the cleavage reaction by EcoRI in the presence of Mn^2+^ *in crystallo*, suggested that the attacking nucleophile is another water molecule close to the water molecule bound to the Mn^2+^, one per subunit (301). All of these candidates for the general base have unfavorable p*K_a_* values, but those of ionizing groups at catalytic centers often deviate by several units from their values in free solution. There is also uncertainty about the extent to which a general base is needed. If the mechanism is not always associative (involving a penta-covalent transition state), but instead is sometimes dissociative (involving a trigonal transition state; Figure 10), then water activation becomes less important, and transition state stabilization becomes very important (317).

**Figure 11.**
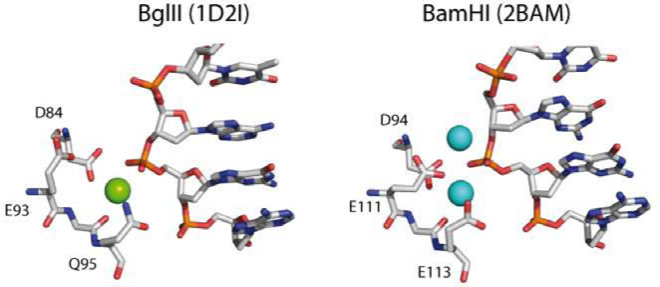
Comparison of the active sites of two structurally very similar restriction enzymes BglII (PDB 1D21, with one Na+ ion in the active center, which can be replaced by a Ca^2+^ ion by soaking) and BamHI (PDB 2BAM, with two Ca^2+^ ions in the active center) (313).

It is also unclear which entity is responsible for protonation of the leaving group. A likely candidate is a water molecule from the hydration sphere of the metal ion cofactor, but the leaving group could also be stabilized by association with a Mg^2+^ ion. Because of the superficial similarities of the active sites of PD…D/EXK enzymes, it is tempting to assume that they all operate in the same way, but the reaction mechanisms of different REases could be similar in some respects, but differ in others. One difference relates to the number of Mg^2+^ ions. As noted by Warshel *et al.* (318): ‘*The detailed mechanism of DNA hydrolysis by enzymes is of significant current interest. One of the most important questions in this respect is the catalytic role of metal ions such as Mg^2+^. While it is clear that divalent ions play a major role in DNA hydrolysis, it is uncertain what function such cations have in hydrolysis and why two are needed in some cases and only one in others*’*.* The question of how many Mg^2+^ ions are involved in catalysis is still unanswered because different numbers of divalent metal ions (often Ca^2+^ instead of Mg^2+^, to avoid cleavage) are found in the co-crystal structures of different REases with their substrates or products (319). In EcoRI and in BglII (313), only a single metal ion is found at the active site; in EcoRV and BamHI (320), there are two (Figure 11).

Different numbers of metal ions are also found in different crystal forms of the same REase–DNA complex. In some co-crystal structures, there are two metal ions in one subunit and none in the other (281,320), or two metal ions in three different locations (321). For EcoRI it was shown that a single Mn^2+^ ion participates in the cleavage reaction *in crystallo* (301). The question arises, then, whether the number of metal ions seen in co-crystal complexes—particularly when these are Ca^2+^—accurately reflect the number of Mg^2+^ ions needed for catalysis. Given these ambiguities one cannot decide how many Mg^2+^ ions are required for DNA cleavage by REases. And also, whether these enzymes all follow exactly the same mechanism for phosphodiester bond hydrolysis (8).

In a recent systematic study of several REases, among them EcoRI and BamHI, which were assumed to follow a mechanism involving exclusively one Mg^2+^ ion or exclusively two Mg^2+^ ions, all were found to exhibit similar Mg^2+^ (or Mn^2+^) concentration dependence, and similar kinetics in response to the presence of Ca^2+^ in addition to Mg^2+^. This study concluded that Type II REases generally have two Me^2+^ binding sites per active center: a high-affinity site (site A), where a Mg^2+^ or Mn^2+^ ion is required for cleavage, and a low-affinity site (site B) which is inhibitory when occupied by Mg^2+^ or Mn^2+^, but stimulatory when occupied by Ca^2+^ at low concentration. Thus, one Mg^2+^ or Mn^2+^ is critical for REase-activation, and binding of a second Me^2+^ modulates this activity. These conclusions are supported by molecular dynamics simulations, and they are consistent with the structural observations of both one and two Me^2+^ ion binding in these enzymes (322). The study also suggested that the essential Mg^2+^ ion might move from site A to site B during catalysis. In a very recent paper with the suggestive title ‘One is enough: insights into the two-metal ion nuclease mechanism from global analysis and computational studies’ results of experiments were published that collectively support a mechanism in which only one metal ion is necessary for nucleic acid hydrolysis by REases (323). We conclude from these studies, then, that there is no general consensus on the number of Mg^2+^ ions involved in catalysis.

### Variations on a theme: subtypes of Type II restriction enzymes

Early investigations of Type II REases focused on EcoRI and EcoRV, but it became clear as more such enzymes were discovered that there were marked differences among them. They were not all cut from the same cloth, so to speak, not even near. Even among enzymes with comparable activities, such as EcoRI (G|AATTC) and BamHI (G|GATCC), or XmaI (C|CCGGG) and SmaI (CCC|GGG), little similarity was found at the amino acid sequence level. This diversity came as a surprise to many investigators, and there is still no general agreement what it means, evolutionarily. Given the metabolic adroitness of prokaryotes, their infinite niches, rapid propagation and endless life-span, it seems likely that every evolutionary scenario possible has had a hand in shaping what we see today, possibly independently many times, in many places. Among Type II REases, compelling examples of convergent evolution abound (e.g. HaeIII, NgoPII and BsuRI (171)), as too do compelling examples of divergent evolution (e.g. Bsu36I, BplI, Bpu10I and BbvCI (324)), neutral drift (e.g. EcoRI and RsrI) and perhaps mosaicism (EcoRI, MunI and MluCI). Examples of gene fusion, separation and exchange are common. All this attests to the genetic resourcefulness of prokaryotes, and to the viral assaults they endure.

There are several ways to bring order to this variety. The conventional way of grouping by genotype (i.e. phylogenetic proximity) is impractical because many Type II REases show no more similarity to one another than do proteins chosen at random. Alternately, REases can be grouped by phenotype based on their behavior and cleavage properties. This is the approach used in the current classification scheme proposed by Roberts and adopted by consensus a decade ago (14). Accumulating information, and improved understanding in the interim, has revealed weaknesses in this scheme, and it will likely be revised in the not-too-distant future. Other ways of classifying Type II REases include grouping enzymes whose structures or subunit/domain organizations are similar (268), or whose catalytic sites are of similar kinds. A discussion of some of these groupings follows.

#### Grouping enzymes by behavior

**Figure 12.**
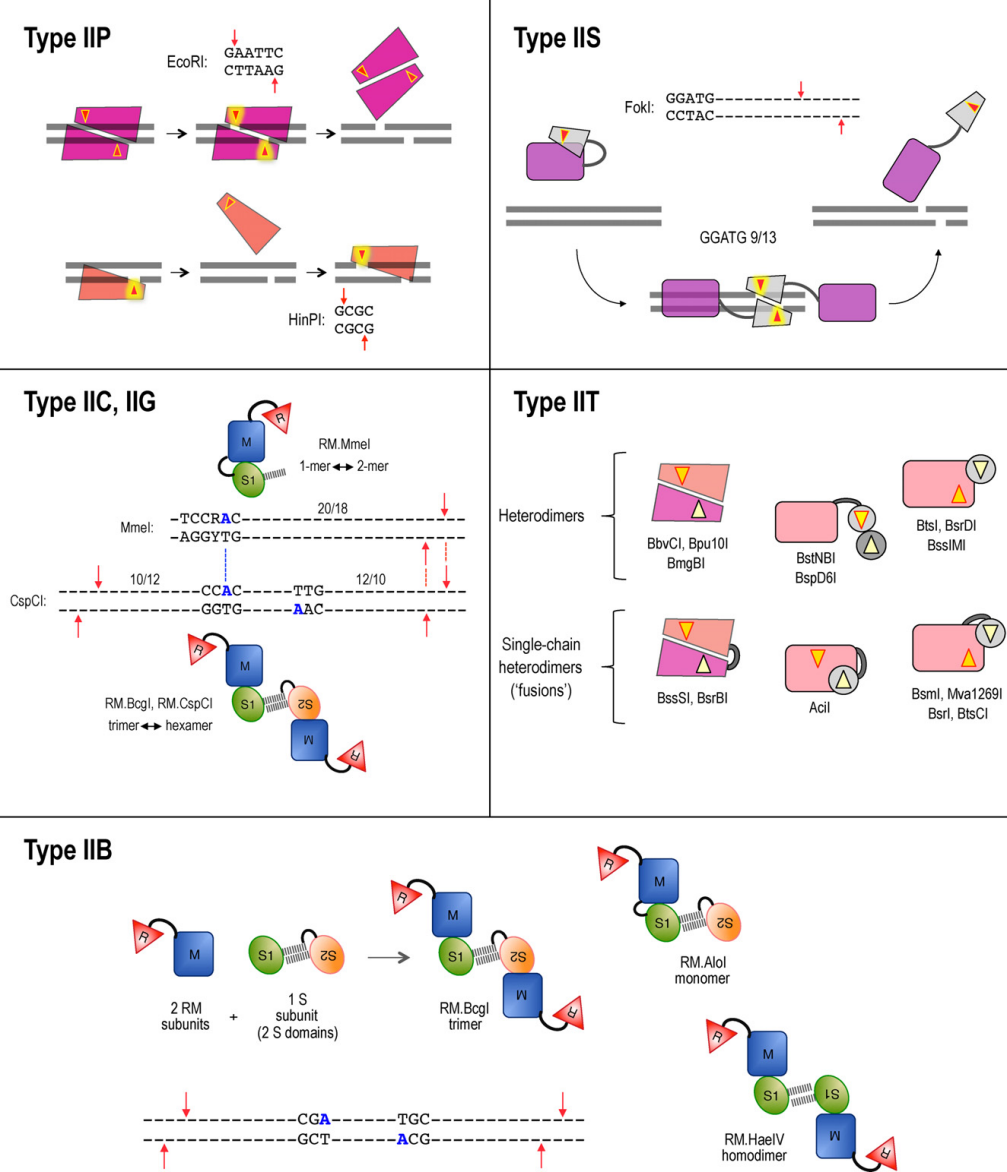
The subunit composition and cleavage mechanism of selected subtypes of Type II REases. *Type IIP* enzymes act mainly as homodimers (top), and cleave both DNA strands at once. Some act as dimers of dimers (homotetramers) instead, and do the same. Still others act as monomers (bottom) and cleave the DNA strands separately, one after the other. Bright triangles represent catalytic sites. *Type IIS* enzymes generally bind as monomers, but cleave as ‘transient’ homodimers. *Type IIB* enzymes cleave on both sides of their bipartite recognition sequences. Their subunit/domain stoichiometry and polypeptide chain continuity varies. Three examples of primary forms are shown: BcgI, AloI and HaeIV. These forms assemble in higher-order oligomers for cleavage. Type IIB enzymes display bilateral symmetry with respect to their methylation and cleavage positions. It is not clear whether they cleave to the left or to the right of the half-sequence bound. *Type IIG* enzymes (e.g. BcgI) might cleave upstream (left) of their bound recognition half-site. All other Type IIG enzymes (e.g. MmeI) cleave downstream from the site, often with the same geometry. These proteins have very similar amino acid sequences, however, suggesting that somehow the reactions are the same. *Type IIT* enzymes cleave within or close to asymmetric sequences. Composition varies; they have two different catalytic sites: top-strand specific and bottom-strand specific. In some, both subunits/domains interact with the recognition sequence (left cartoons). In others, only the larger subunit/domain recognizes the DNA.

In the survey and nomenclature of Type II REases by Roberts *et al.* (14), 11 subtypes were defined each with a particular, but not necessarily unique, property: A, B, C, E, F, G, H, M, P, S and T. EcoRI, EcoRV and most of the familiar laboratory cloning enzymes belong to the so-called ‘IIP’ subtype because they recognize **p**alindromic (symmetric) DNA sequences. In this classification scheme, subtypes are not mutually exclusive, and enzymes can belong to several subtypes at once. FokI, for example, is perhaps the best-known member of the ‘IIS’ subtype (**s**hifted cleavage), but it also belongs to the ‘IIA’ subtype because its recognition sequence is **a**symmetric. And BcgI, an extreme example, belongs to six, and arguably more, subtypes (325). Supplementary Table S3 gives an overview of the occurrence of the subtypes among characterized REases, as summarized in REBASE. Figure 12 shows schematically the subunit composition and cleavage processes of selected subtypes.

#### Type IIA

Type IIA enzymes, for example SapI (GCTCTTC 1/4), recognize **a**symmetric sequences and cleave within, or a defined distance away from, the sequence. Many are accompanied by one or two DNA methyltransferases (MTases) that each modify one strand of the recognition sequence. Others are combination restriction *and* modification (RM) enzymes, some of which have separate accompanying MTases, while others do not. Asymmetric recognition sequences occur more frequently in DNA than do symmetric sequences and so, *a priori*, one might expect Type IIA REases to be more abundant than Type IIP. This is not the case, however. Type IIP enzymes recognize their sequences overwhelmingly as homodimers—this being the most efficient use of genetic resources—and so they are more common than they would otherwise be.

The fundamental challenge for REases of all types is that the reaction they catalyze is polarized and strands of DNA have opposite polarities. Only one reaction trajectory produces 5-phosphate, 3-hydroxyl ends. To generate these on both strands requires either that the catalytic site works in both directions, or that it adopts the opposite orientation for each strand. If it can work in both directions then, in principle, the same site could cleave both strands, one after the other, without switching orientations. If it can work in only one direction, however, it must swivel 180° between strands. And if it cannot swivel, then either the entire enzyme must detach, rotate and reattach—available only to REases with symmetric specificities—or two catalytic sites in opposite orientations must be present in the enzyme to begin with. The symmetric catalytic site of BfiI (ACTGGG 5/4), formed at the subunit interface of a homodimer, was for a time thought to act bi-directionally (326), but now it is believed to swivel, instead (327). No other REase catalytic site is known to swivel, and none are known to act bi-directionally, although examples might well be found eventually.

Type IIP enzymes take advantage of dimerization for DNA cleavage as well as for recognition by using two copies of the same catalytic site to juxtapose the two DNA strands. Most Type IIA enzymes are thought to dimerize too, in order to cleave, but only briefly. The recognition and cleavage components of Type IIA enzymes are usually segregated into different domains. The recognition domain binds to DNA individually and asymmetrically, but the cleavage domain is thought to dimerize with an identical domain from another molecule for cleavage. Evidence for this ‘transient dimerization’ (328,329) comes mainly from kinetic studies which indicate that cleavage is cooperative. With these enzymes, cleavage rates often increase disproportionately with increasing enzyme concentration, and are usually higher on substrates with multiple recognition sequences than on substrates with single recognition sequences. Not all Type IIA REases dimerize transiently, however. Some use two different catalytic sites instead, from different subunits (e.g. BbvCI) or from different domains within the same protein chain (e.g. Mva1269I). These enzymes tend to cleave within the recognition sequence or very close to it, at positions inaccessible to separate dimeric catalytic domains.

#### Type IIB

Type IIB enzymes (reviewed by Marshall and Halford (325)), for example BcgI (10/12 CGA N6 TGC 12/10), cleave DNA on **b**oth sides of their recognition sequence, releasing a small (e.g. 34 bp) fragment that contains the recognition sequence. They are large, complex, RM enzymes that methylate DNA in addition to cleaving it. They function alone, without accompanying MTases. Type IIB recognition sequences are bipartite, comprising two specific ‘half-sites’ separated by a short non-specific gap. The enzymes are related to, and share many features in common with, Type I REases (see Loenen *et al.* for a comparison (1)). Their catalytic site for cleavage belongs to the PD-D/EXK superfamily (330) and forms the N-terminal domain of the RM protein. Cleavage produces 3′-overhangs of two to five bases, suggesting that the catalytic sites juxtapose across the minor, rather than the major, DNA groove. Their catalytic site for methylation belongs to the gamma-class (NPPF/Y/W) family; it lies distal to the cleavage domain in the RM protein and methylates adenine residues. Methylation converts one A in the top strand of the first (5′) half-site to *N*6-methyladenine (m6A) and one A in the bottom strand of the second (3′) half-site. AdoMet is required for methylation and also, in some enzymes, for cleavage (331).

In principle, when Type IIB enzymes encounter a recognition sequence, they have three options: they can ignore the sequence (neutral mode); they can cleave it (restriction mode); or, they can methylate it (modification mode). To act appropriately according to circumstances requires some sophistication (332,333). How these alternative modes are implemented is not clear, but the signal is likely to be the methylation states of the recognition half-sites. If both half-sites are methylated ( = fully modified host DNA), the sequence should be ignored. If only one half-site is methylated ( = newly replicated host DNA), the other must be re-methylated. And if neither half-site is methylated ( = potentially foreign DNA), the enzyme must refrain from methylation, and either cleave the sequence immediately or wait until its significance becomes clearer. The simplest, restriction-only, Type IIP enzymes such as EcoRI and BamHI cleave immediately they encounter an unmethylated recognition sequence, but Type IIB enzymes, and many others, in effect assess the situation first. If multiple sequences are unmethylated ( = verified foreign DNA), the DNA is cleaved. But if only one is unmethylated ( = inadvertently unmodified host DNA), cleavage is suppressed, and the sequence is eventually re-methylated. All of these steps involve sensing, subunit intercommunication and catalytic adaptation to suit.

BcgI, CspCI and BsaXI possess separate sequence-specificity (S) subunits that, like Type I S-subunits, comprise two opposed sequence-recognition domains, one for binding each half-site. The biochemistry of BcgI, and of other Type IIB enzymes, has been studied in depth by Stephen Halford's group at Bristol University. BcgI has the subunit stoichiometry 2RM:1S, in which one RM catalytic subunit associates with one sequence-recognition domain (334). It exists as a hetero-hexamer—a dimer of trimers (2× [2RM + 1S])—in solution and also when bound to DNA (335). In restriction mode, BcgI is active only when bound to two recognition sequences, whereupon cleavage of all four double strands occurs at once (333,336). This involves hydrolysis of eight phosphodiester bonds and requires four additional catalytic sites that are thought to be contributed by neighboring enzyme molecules, or by surplus individual RM subunits. In modification mode, BcgI binds single sequences also as a hexamer. It methylates hemimethylated sequences rapidly, but unmethylated sequences are methylated 100-fold less efficiently. BcgI is thus far more of a ‘maintenance’ MTase than a ‘de novo’ MTase, and is in fact the most extreme prokaryotic example known (332). These cleavage and methylation properties of BcgI are consistent with the ‘wait and see’ mechanism discussed above.

Other Type IIB REases, including AloI, PpiI (337) and CjeI, are single-chain proteins, in which the specificity portion, again comprising two sequence-recognition domains, forms the C-terminus of a composite, single-chain protein of composition RMS. In these enzymes, subunit fusion results in an imbalance in the number of catalytic and specificity components. It is not entirely clear how these proteins function, with only one catalytic domain to share between two recognition half-sites. Recent study of the single-chain enzyme, TstI, indicates that it acts as a homotetramer bound to two bipartite recognition sequences rather than four, and behaves in a different fashion to BcgI in both cleavage and methylation (338). As with Type I RM enzymes, the individual sequence-recognition domains of Type IIB enzymes function independently, and can be ‘swapped’ for one another to generate enzymes with new combinations of recognition sequence specificities (337). Most Type IIB enzymes are inactive as endonucleases when bound to single recognition sites (325,331), and active when bound to two sites preferably in *cis* on the same DNA molecule, or in *trans* on concatenates. To cleave multiple DNA duplexes at once requires these proteins to assemble into large oligomers with molecular masses in excess of 500 kDa, making them the largest REases known (333). None of the Type IIB enzymes have been crystallized. Much remains to be learned about them, and it is clear that they are not straightforward.

#### Type IIC

Type IIC enzymes (**c**ombined) have endonuclease and methyltransferase activities in the same protein. The majority comprise an N-terminal PD-D/EXK endonuclease domain followed by a gamma-class (NPPY/F/W) methylation domain, and they include all the known Type IIB REases mentioned in the previous section. At least one Type IIC enzyme is known that differs: BtgZI (GCGATG 10/14). This REase comprises instead an N-terminal alpha-class MTase, and a C-terminal variant of the PD-D/EXK domain in which glutamine (Q) replaces glutamate (E). Cleavage by BtgZI creates a 4-base, 5′-overhang instead of the usual 3′-overhang. Some Type IIC REases function without a separate MTase (e.g. MmeI). Others, such as Eco57I (339,340), are accompanied by one MTase, and yet others, such as BpuSI, (341) by two MTases. Most Type IIC REases bind to their target sequences as monomers. Some recognize sequences that are continuous and asymmetric, and they cleave on only one side of the sequence approximately one turn of the helix away (e.g. Tth111II; CAARCA 11/9), one and one-half turns away (e.g. Eco57I: CTGAAG 16/14), or two turns away (e.g. MmeI (TCCRAC 20/18)). Others, including the single-chain Type IIB enzymes such as AloI (7/12 GAAC N6 TCC 12/7), have two different sequence-specificity domains, and so their recognition sequences are bipartite and asymmetric, and they cleave on both sides (342). A few, such as HaeIV (7/13 GAY N5 RTC 14/9), bind as homodimers using two copies of the same specificity domain (343). Their recognition sequences are also bipartite, but symmetric, and they also cleave on both sides. HaeIV makes the first strand cleavage randomly on either side of the recognition sequence; the second strand cleavage occurs more slowly (343).

Among Type IIC enzymes, the crystal structure of BpuSI (GGGAC 10/14) has been solved, but without DNA (344). The structures of MmeI and NmeAIII have also been solved recently (345), the former with DNA, and are awaiting publication. Type IIC RM proteins possess only one endonuclease catalytic site, yet cleave both DNA strands. Cleavage is presumed to involve ‘transient dimerization’ between the catalytic domains of neighboring molecules, as has been proposed for FokI (328,329). For many Type IIC enzymes, cleavage efficiency increases when the substrate DNA contains multiple recognition sites, or when oligos containing recognition sequences are added. Multiple sites are thought to raise the local enzyme concentration and thereby enhance transient dimerization, and specific binding is thought to render the catalytic domain competent to dimerize. Type IIC enzymes cleave away from their recognition sites, and the distance can vary by ±1 or 2 bp. The ‘reach’ between the recognition sequence and the cleavage sites is thought to depend upon physical distance rather than the number of intervening base pairs, and this can vary according to DNA topology, ionic conditions and base pair sequence. For enzymes that cut on both sides of bipartite recognition sequences, when the reach is measured from the adenine that becomes modified in each half-site rather than the boundaries of the recognition sequence, it is typically the same on the left as on the right, because the same protein catalyzes both reactions on both sides.

#### Type IIE

The simplest Type II REases such as EcoRI and BamHI cleave DNA efficiently regardless of the number of recognition sites present in the substrate molecule. EcoRII (|CCWGG; (47,50)), discovered shortly after EcoRI, behaves differently and requires multiple sites for efficient cleavage. EcoRII acts as a homodimer and binds to two (346) or three (347,348) copies of its pseudo-palindromic recognition sequence at once. Like other homodimers, one sequence is bound concertedly by the two subunits, in the normal DNA-recognition groove between them (349). This sequence becomes cleaved, and it is bound by the C-terminal domains of the EcoRII subunits, which contain the catalytic sites. (Surprisingly, it was found that the central A and T (W) bases of the CCWGG sequence are flipped out from the helix by EcoRII, and by similar enzymes such as PspGI (|CCWGG) and Ecl18kI/SsoII (|CCNGG), compressing the recognition sequence in effect to just CC-GG (90).) The other sequences are bound in a different way by EcoRII, without cleavage, by the individual N-terminal domains that act as allosteric activator(s) or effector(s) (349,350). Type IIE REases are Type IIP enzymes with allosteric **e**ffector domains that stimulate catalysis when bound to additional recognition sequences (reviewed by Mucke *et al.* (351)). NaeI (GCC|CCG), another example of a Type IIE REase (229,352), has an intriguing functional connection to DNA topoisomerases (353,354), and, like EcoRII, it induces looping in bound DNA (355), and it can be stimulated by oligos containing its recognition sequence to cleave otherwise refractory sites (356). The properties of loop formation, and stimulation by the addition of specific oligos, are typical of these enzymes. The biological function of allosteric activation is unclear, but it might be to spare the cell's own DNA from cleavage at inadvertently unmodified sites during periods when DNA methylation fails to keep up with DNA synthesis. The same might also pertain to the Type IIF enzymes described below.

#### Type IIF

Type IIF enzymes [reviewed by Siksnys *et al.* (357)] bind two recognition sequences and cleave coordinately, hydrolyzing all **f**our DNA strands at once. Type IIE REases also act by binding two (or more) sequences, but they differ from Type IIF enzymes in that when they do, only one of the sequences is cleaved because their binding sites differ: one is solely catalytic, the other(s) solely allosteric. Type IIF REases, in contrast, act as homotetramers; their binding sites are identical and catalytic, but they have allosteric, or ‘cooperative’, properties, too. Some of these REases, such as the Type IIB TstI mentioned above and the Type IIS BspMI (358), recognize asymmetric sequences with structural organizations that remain uncertain. Others, such as SfiI (GGCCNNNN|NGGCC) (359), the related REases Cfr10I/Bse634I (R|CCGGY) and NgoMIV (G|CCGGC) (360–362), and possibly PluTI (GGCGC|C) (363), recognize symmetric sequences as pairs of ‘back-to-back’ homodimers. The binding site of each homodimer is catalytic, like those of ordinary homodimers, but unable to cleave unless the other binding site is also occupied. Both binding sites must be occupied, then, for either to be active and when they are, both sequences cleave at once (360,364). For reasons of stability, binding two sequences in *cis* is preferred to binding in *trans*, and results in looping out of the intervening DNA (365). The way in which DNA binding is signaled between the catalytic sites is unclear, but likely involves conformational changes that propagate across the tetramer interface (362). In Bse634I, mutation at this interface results in homodimers that bind and cleave single recognition sequences efficiently, indicating that tetramerization is inhibitory (362). A mutation at the tetramer interface of SfiI also relieves inhibition by allowing the enzyme to bind to a single sequence and cleave it efficiently while remaining a tetramer (128).

SgrAI (CR|CCGGYG) belongs to the same enzyme family as Cfr10I/Bse634I and NgoMIV. It is more active on substrates with two recognition sequences than one, and cleaves both sequences concertedly (366). SgrAI also assembles into homotetramers, but then goes further and forms ‘run-on’ oligomers comprising helical filaments of one DNA-bound homodimer after another. Adjacent homodimers are offset ∼90°, rather than back-to-back, and four homodimers together form almost one turn of a left-handed spiral, which can comprise up to 18 homodimers and possibly more (367). In this oligomeric form, SgrAI is highly active on both its canonical sequence and on a ‘star’ sequence, CR|CCGGYN (N = any base). The allosteric effect is thought to stem from interactions between the minor groove of the DNA flanking the recognition sequence and protein loops to the side of the binding site. Cleavage of star sequences is less efficient (4%) than cleavage of the canonical sequence (368), but it is much higher than occurs with most other REases. It implies that the SgrAI homodimer is somewhat asymmetric, such that one subunit consistently recognizes the outer base pair of the recognition sequence, while the other subunit sometimes does not. The homodimer undergoes significant conformational adjustments when it assembles into oligomers (367), and these changes might introduce asymmetry with respect to sequence recognition.

Related REases can act as either Type IIE or IIF enzymes. EcoRII (|CCWGG; Type IIE) and Ecl18kI/SsoII (|CCNGG; Type IIF), for example, interact with their recognition sequences as homodimers, and use similar base-recognition, base-flipping and cleavage mechanisms. The two enzymes are structurally similar except for the N-terminal allosteric effector domain of EcoRII, which is not present on Ecl18kI/SsoII. Nevertheless Ecl18kI/SsoII, like EcoRII, is more active on substrates with multiple recognition sites than on substrates with single sites, suggesting that binding to two sites is required for DNA cleavage. Instead of possessing a dedicated effector domain like EcoRII, however, Ecl18kI/SsoII assembles into a ‘transient’ tetramer to accomplish cleavage (369). Evolutionarily diverged versions of the same enzyme can also act in different ways. Cfr42I and Eco29kI, for example, recognize and cleave the same DNA sequence, CCGC|GG, and have similar GIY-YIG catalytic sites (242,370). Cfr42I is a tetramer in solution, binds to two recognition sequences, and cleaves both sequences at once (21). Eco29kI, in contrast, purifies as a monomer in solution (371), but binds to its recognition sequence as a homodimer, and cleaves one recognition sequence at a time (372); Eco29kI also crystallizes with DNA as a homodimer (239); Cfr42I has not been crystallized.

#### Type IIG

Type IIG REases are stimulated by, or absolutely require, AdoMet. Most of the Type IIB and Type IIC enzymes are of this kind, and the group as a whole is referred to loosely as ‘Type IIG’. These are combined RM enzymes, with a DNA-cleavage domain and a **g**amma-class DNA-methylation domain in a single protein chain. Both R and M catalytic activities are harnessed to the same sequence-specificity module (S), which can occur as a separate subunit or as the C-terminus of the RM protein. S-modules can recognize single (continuous) DNA sequences, or bipartite (discontinuous) sequences, either of which can be symmetric or asymmetric. Type IIG enzymes occur in a variety of oligomeric forms, with or without separate, accompanying MTases. A summary of Type IIG REase organizations, and of their relationship to Type I REases and certain Type II MTases, is given in Loenen *et al.* (1). AdoMet is the donor of the methyl group, and so it is essential for the methylation reaction. Since it also either stimulates, or is absolutely required for, the cleavage reaction, it likely acts as an allosteric activator, too. The advantage of AdoMet dependency again might be self-preservation, since it reduces the likelihood that the cell's own DNA will be cleaved at times of AdoMet shortage and consequent undeR–Modification. Since Type IIG enzymes methylate as well as cleave DNA, *in vitro* digestions often fail to go to completion. A proportion of sequences become modified during incubation, and thereafter are resistant to cleavage (338).

The crystal structure of BpuSI (GGGAC 10/14) has been solved without bound DNA (344). The enzyme comprises an N-terminal endonuclease domain, a central gamma-class methyltransferase domain, and a C-terminal specificity domain. Structural comparisons and modeling show that in order to bind DNA specifically, the C-terminal domain of BpuSI must rotate with respect to the R and M domains, and reorganize. Large rearrangements often accompany DNA binding by REases, as can be seen by comparing the crystal structures of unbound and specifically bound forms of, for example, MvaI (pdb:2OA9 and 2OAA (131)), EcoO109I (pdb:1WTD and 1WTE (315)) and HinP1I (pdb: 1YNM and 2FKC (130,373)).

If the DNA sequence recognized by Type IIG enzymes changes—by mutations in the S-module (374), for example, or by domain exchange (337)—it does so for both restriction and modification activities in the same way at the same time. This functional synchrony has allowed the specificities of certain Type IIG enzymes, such as those of the MmeI-family, to diverge widely in the course of evolution. Numerous MmeI-family enzymes have been characterized, each similarly organized and similar in aa sequence and hence structure, but specific for a different 6–8 bp recognition sequence ((375) and Supplementary Table S1, group E). The C-alpha backbone of the recognition domain of these proteins has evolved a conformation that allows different pairs of amino acids to specify alternative base pairs in the sequence recognized. Thus E806…R808 (Glu…Arg) in MmeI (TCCRA**C** 20/18) specifies C at the last position of the recognition sequence, whereas K806…D808 (Lys…Asp) specifies G, instead (i.e. TCCRA**G**) (374). Other pairs of amino acids within the specificity domain determine other base pairs in the recognition sequences. This is unusual behavior for restriction enzymes, which as a whole have evolved in the other direction, toward recognition sequence immutability, instead (313).

Because the sequences recognized by MmeI-family enzymes are generally asymmetric, only one DNA strand becomes methylated—always the invariant adenine in the ‘top’ strand: TCCR**A**G, in the case of MmeI (376). When such hemimethylated sequences replicate, one daughter duplex retains the hemimethylation, but the other becomes completely unmethylated. How unmethylated daughter sequences are distinguished from foreign DNA is unclear, but it seems likely that pairs of sequences in opposite orientations, and perhaps several pairs, are monitored before the enzyme commits to either cleavage or re-methylation.

MmeI-family enzymes cleave substrates with multiple sites more efficiently than substrates with single sites, and cleavage is stimulated by the addition of oligos that contain a recognition site. The enzymes purify as monomers, but there are strong indications that they cleave as homodimers (or higher-order oligomers) formed between enzyme molecules bound to adjacent, opposed recognition sites. When modeled, the structures of these complexes closely resemble Type I REases, with the difference that Type IIG cleavage domains cut DNA at fixed positions close to their recognition site(s) whereas Type I R-subunits cleave at variable distances, far away. Attempts to harmonize the cleavage behavior of MmeI (TCCRAC 20/18) and Type IIB enzymes such as BcgI (10/12 CGA N6 TGC 12/10) that are organizationally similar suggest that when these proteins bind to their recognition site(s), their endonuclease domains might cleave, not the DNA at the site to which they are bound, but rather the DNA at the *other* site, instead. Unless we misread the situation, enzymes of this kind perform some interesting gymnastics in the course of their cleavage reactions (333,338). The catalytic complexes of Type IIG enzymes are likely to be large and difficult to solve by crystallography. Alternative approaches such as single particle cryo-electron microscopy and reconstruction (367,377,378), or molecular modeling (379), might prove fruitful in the interim.

Most prokaryotes encode no more than one or two Type IIG REases, along with a variety of Type I, Type IIP, Type IIS and, less frequently, Type III enzymes. Extreme differences can be found, however. *Helicobacter pylori* isolates tend to have large numbers of Type IIP and IIS systems—up to 20—and four to six Type IIG systems. *Borrelia burgdorferi* isolates, in contrast, can have up to 20 Type IIG systems, to the complete exclusion of all other types. We know little about the selective advantages and disadvantages that underlie these variations.

#### Type IIH

When the AhdI R–M system was analyzed, it was found to comprise a Type IIP-like REase (GACNNN|NNGTC) and an unusual accompanying MTase (G**A**C N5 GTC; ‘**A**’ = position of m6A-methylation). The M.AhdI MTase consisted of a catalytic subunit for methylation (M) and a separate specificity subunit (S) for DNA sequence recognition, and it acted as a 2M+2S tetramer, an organization suggestive of ancestral Type I MTases (380). AhdI was colloquially referred to as a ‘Type $1\frac{1}{2}$’ R–M system because it was a ‘missing-link’ in the evolutionary chain, part Type I and part Type II. ‘Type $1\frac{1}{2}$’ was informal, and so Type IIH (**h**ybrid) was adopted instead. We now know that in addition to forming the core of all Type I and most Type IIG REases, the gamma-class adenine-MTases (the NPPY/F/W group), of which M.AhdI is one, are widespread and adaptable, and accompany many Type II REases, both those recognizing continuous sequences (e.g. M.TaqI: TCG**A**; HincII: GTYR**A**C) and those recognizing bipartite sequences (e.g. M.DrdI: G**A**C N6 GTC; M.XcmI: CC**A** N9 TGG). The Type IIH distinction seems less important, now, as also do several of the other Type II sub-classifications, and it is rarely used.

#### Type IIM

Type IIM enzymes require **m**ethylated recognition sequences. The best-known example is DpnI (Gm6A|TC), discovered by Lacks and Greenberg (381). DpnI acts as a monomer and cleaves its recognition sequence one strand at a time, as do several other Type II REases with short recognition sequence, such as HinP1I (373), and BcnI (132). DpnI consists of an N-terminal catalytic PD-D/EXK domain, and a C-terminal winged helix (wH) allosteric activator domain. Both domains bind DNA in a sequence- and methylation-dependent manner. DpnI has been crystallized with DNA bound at the C-terminal effector domain, but not at the catalytic domain (24). DpnII, an allelic alternative to DpnI *in vivo*, cleaves the same GATC sequence, but only if it is *un*methylated (382). The complementary specificities of DpnI and DpnII proved to be very useful for site-directed mutagenesis experiments. It remains unclear what structural features of DpnI account for its absolute dependence on methylated adenines within its recognition sequence. Methyl groups can increase the affinity between a protein and a DNA sequence through hydrophobic interactions, but this will hardly produce the all-or-nothing behavior seen among the methylation-dependent REases. It is possible, instead, that the methyl groups induce a structural change by, for example, altering the side-chain conformations of long-chain amino acids such as arginine and lysine, switching them from conformations that interfere with and prevent binding, to conformations that are compatible with and permit binding.

#### Type IIP

Type IIP enzymes are the most ubiquitous and varied of the Type II REases. They recognize symmetric (**p**alindromic) sequences and cleave symmetrically within the sequence (e.g. EcoRI: G|AATTC) or, less often, at its boundaries (e.g. EcoRII: |CCWGG). Almost always, Type IIP REases are accompanied by one, and in rare cases two, separate MTases of identical sequence specificity. Some Type IIP REases act as monomers, but most act as homodimers or homotetramers, and this structural duplication accounts for their symmetry in specificity and catalysis. The multimers generally, but not always (383), cleave both strands of the DNA duplex in the same binding event. The monomers cleave DNA one strand at a time, but without the release of nicked intermediate, indicating that the same enzyme *molecule* cleaves both DNA strands at each recognition sequence (133), first one strand and then the other. Since these strands have opposite 5′ to 3′ orientations, and the catalytic reaction is polarized, monomeric REases must dissociate from the recognition sequence after the first cleavage, rotate 180°, and then re-associate in the opposite orientation in order to cleave the second strand. This they do without detaching from the DNA and returning to bulk solution (133). A careful analysis of the reaction pathway of the monomer BcnI (CC|SGG) showed that in a rapid first step the enzyme hydrolyzes either strand, with a small preference for the ‘G-strand’ (CCGGG) over the ‘C-strand’ (CCCGG); in a slow second step it slides away, rotates, and then returns to the sequence in the other orientation; and in a rapid final step it hydrolyzes the other strand. Much of what we know about the monomeric Type IIP REases comes from synergistic collaborations between Virginijus Siksnys’ biochemistry group and Matthias Bochtler's crystallography group.

Type IIP recognition sequences are usually 4–8 specific base pairs in length. They can be continuous (e.g. HindIII: A|AGCTT) or discontinuous, with one internal non-specific base pair (e.g. HinfI: G|ANTC), two (e.g. Hpy188III: TCN|NGA), three (e.g. DraIII: CACNNN|GTG), four (e.g. XmnI: GAANN|NNTTC), five (e.g. BglI GCCNNNN|NGGC) or more, up to a record nine (e.g. XcmI: CCANNNNN|NNNNTGG), depending on the geometric relationship between the two subunits in the homodimer. Cleavage can produce flush ends, or it can be staggered and produce 5′- or 3′-overhangs of 1, 2, 3 or 4 bases, and occasionally more. Recognition sequences can comprise a single base pair (e.g. XmaI: C|CCGGG; DraI: TTT|AAA), or both base pairs, and many enzymes can accommodate alternative base pairs at certain positions such as R:Y (pu**r**ine:p**y**rimidine = A:T or G:C), W:W (**w**eak base-pairing = A:T or T:A), S:S (**s**trong base pairing = G:C or C:G) and M:K (**m**ethylatable base = A:T or C:G) among others.

Hundreds of different Type IIP specificities are known. For each, usually several, and sometimes very many, REases of identical specificity and similar amino acid sequence can be found in other bacteria and archaea. These ‘isoschizomers’ often represent diverged versions of the same ancestral enzyme, the gene of which has moved laterally between prokaryotes and accumulated neutral changes over time. Even among related enzymes, significant differences in biochemical behavior have been noted (383). Often, clusters of REases with closely related specificities display clear amino acid sequence similarity—PstI (CTGCA|G) and SbfI (CCTGCA|GG), for example, or BssHII (G|CGCGC) and AscI (GG|CGCGCC)—signifying recent radiation from a common ancestor. REases with unrelated specificities generally display no amino acid sequence similarity, however, signifying either that no trace of common ancestry remains due to the passage of time, or that they arose independently to begin with. Aside from the hundreds of different Type IIP REases that have been characterized, the genes for thousands more have been identified by bioinformatics analysis of sequenced microbial genomes (see REBASE/REBASE Genomes for a current compilation). These encode ‘putative’ (i.e. unverified) REases consisting of isoschizomers, and likely novel enzymes with related, but as yet undiscovered, specificities. Type IIP REases are symmetric, relatively small, and the least difficult to crystallize. Most of the REases that have been crystallized with substrate DNA belong to the IIP subtype, around 35 enzymes in all (Supplementary Table 2).

#### Type IIS

By definition, Type IIS REases cleave DNA at fixed positions outside of their recognition sequence. Cleavage is **s**hifted to one side of the sequence, within one or two turns of the double helix away. Type IIS enzymes were first discerned as being different by Waclaw Szybalski and colleagues at the University of Wisconsin (384), who devised a variety of ingenious applications for them (385,386). FokI, one of the earliest such enzymes discovered (387), is the best known and is the source of the DNA-cleavage domain used in synthetic gene-targeting endonucleases (388). Technically, all Type IIB, C and G REases (e.g. BcgI, Eco57I, MmeI) are Type IIS enzymes, too, because they cleave outside of their recognition sequences. These form a close-knit group centered on their core gamma-class MTase domain, as described above. They are distinct from the rest of the Type IIS enzymes, and are excluded from the discussion that follows. FokI has been studied in some depth, and has been crystallized with and without bound DNA (262,328–329). Apart from BfiI, which is very unusual (327), few other Type IIS REases have been studied in detail and, for want of better understanding, FokI is considered representative of the Type IIS subclass, although other kinds likely exist. In Type IIP REases, the amino acids responsible for recognition and for catalysis are integrated into one composite domain (268). In Type IIS REases, they occur in different domains, which can be split into separate protein chains (389). Type IIS REases are generally larger than Type IIP REases and comprise an N-terminal, sequence-specific recognition domain, a connecting ‘linker’ or ‘arm’, and a C-terminal DNA-cleavage domain with no sequence specificity.

Type IIS recognition sequences are usually asymmetric. In all likelihood this is not through necessity, but rather reflects the fact that far more asymmetric DNA sequences exist to be recognized than symmetric sequences. Because the recognition sequence is asymmetric, cleavage takes place on only one side. If it were symmetric, both sides would become cleaved, first one and then the other. NmeDI (12/7 RCCGGY 7/12) is an example of just such a symmetric Type IIS enzyme (325,390). FokI recognizes 5′-GGATG-3′/5′-CATCC-3′ and catalyzes staggered cleavage 9 bases away on one strand and 13 bases away on the other, producing fragments with 4-base, 5′-overhangs. By convention, the recognition sequence of Type IIS enzymes is written in the orientation in which cleavage occurs to the right of the sequence, downstream of what is then defined as the ‘top’ strand. Thus, the catalytic activity of FokI is written ‘GGATG 9/13’, by convention, rather than the equally accurate, ‘13/9 CATCC’. Type IIS REases are usually accompanied by two separate MTases, each of which modifies one strand of the recognition sequence by methylating one adenine or one cytosine in that strand. Often, these MTases occur as individual proteins, but sometimes, as is the case in the FokI R–M system, they are joined into one protein chain (391,392). The benefits of such fusions are unknown but, all things being equal, it allows the MTases to be synthesized in a fixed, 1:1 ratio and their synthesis to be co-regulated. And, if the hemimethylated daughter DNA duplexes are re-methylated as they emerge from the replication complex, whenever one MTase is needed to service one duplex, the other MTase is on hand to service the other duplex.

A few Type IIS R–M systems include only one companion MTase, rather than two. These systems recognize quasi-palindromic sequences that are viewed as *a*symmetric by the REase, but symmetric (and ambiguous) by the MTase. As a consequence, both strands of the recognition sequence become modified by just the one MTase. Examples include BbvI (REase: GCAGC 8/12; MTase: G**C**WGC) and MlyI (REase: GAGTC 4/4; MTase: G**A**STC). There is a price to be paid for methylation, and prokaryotes go to lengths not to squander it. The Type IIS R–M system AlwI comprises an REase (GGATC 4/5) and two MTases joined into a single chain, one specific for the top strand sequence, GG**A**TC, the other for the complementary bottom-strand sequence, G**A**TCC. A single MTase such as M.MboI, M.DpnII or Dam (G**A**TC) methylates both of these strands at the same positions, and protects the AlwI sequence from cleavage just as effectively as do its two, complementary MTases. However, they also methylate additional sequences (AGATC, TGATC and CGATC) that are not necessary for protection from AlwI, and the evolutionary cost of this has given the two-MTase solution adopted by the AlwI R–M system the selective edge.

FokI consists of an N-terminal DNA-binding domain and a C-terminal, non-specific, cleavage domain (328). Like other Type IIS REases that cleave more than a few base pairs from their recognition sequence, the FokI cleavage domain contains only one catalytic site, in this case of the PD-D/EXK kind. Three important observations came from the FokI structural studies that have guided thinking since not only about Type IIS REases but about Type IIB, C and G REases, too. First, in FokI crystal structures, the catalytic domain is ‘sequestered’ by the DNA-binding domain in a position that is unfavorable for DNA cleavage. This suggests that the catalytic domain might be controlled to prevent non-specific DNA cleavage, and that it is restrained during linear diffusion/three-dimensional hopping, and then released, perhaps due to a conformational change in the DNA-binding domain, when the recognition site is acquired. (The Type IIP REase, SfiI (GGCCNNNN|NGGCC), cleaves within a 5 bp non-specific sequence, and so its catalytic sites also disregard the flanking base pair sequence, much like the FokI catalytic sites. In the crystal structure of SfiI with DNA, the catalytic sites are too far from the DNA to initiate cleavage, exemplifying perhaps another cleavage-control mechanism (393)). Second, nicked DNA intermediate does not accumulate during the FokI cleavage reaction, suggesting that an individual cleavage domain cannot catalyze strand-cleavage on its own. And third, cleavage is stimulated by multiple recognition sites in the DNA, and by the addition of the purified catalytic domain, suggesting that cleavage of duplex DNA requires the dimerization of two catalytic domains (329).

Pieces of the cleavage puzzle are still missing and await further experimentation, but the current idea is that double-strand cleavage by Type IIS REases requires dimerization of the catalytic domains of nearby molecules at least one of which is specifically bound to a recognition site (329,378). In some cases the second molecule can be free in solution or bound to DNA non-specifically (394), but the complex is more stable when it, too, is specifically bound (395,396). If so, the two sites do not have to be nearby, or in any particular orientation, and if they are far apart, DNA looping takes place between them (397,398). The requirement that two enzyme molecules be specifically bound to interact productively for catalysis could be another example of the ‘wait and see’ precaution discussed earlier for Type IIE and IIF enzymes. A surprisingly large number of Type II REases behave in this way, in fact, and require at least two recognition sites in order to cleave (399–401). In the case of FokI, when molecules bound to two sites associate (‘synapse’), the recognition sequences are held side-by-side, in parallel (402). This is somewhat surprising because in order to dimerize, the catalytic domains must assume opposite orientations. It is easier to visualize this happening between molecules that approach one another head-on, than sideways. Ultimately in the synapses, both bound DNA duplexes become cleaved. This suggests that the catalytic domains can shift from one side to the other, cleaving first one duplex and then moving over to cleave the other.

The co-crystal structure of FokI reveals how the DNA interacts specifically with the binding domain (pdb:1FOK), but not how it interacts with the cleavage domain(s) (262). The alternative structure, in which the cleavage domains are dimerized (pdb:2FOK), lacks DNA (328). In fact, for all of the many REases we suppose dimerize transiently through their catalytic domains when they cleave DNA, not a single structure revealing this event has been obtained. Instead we rely on modeling. Modeling suggests that when the FokI catalytic domain releases from its sequestered position in the specific complex, the connecting arm between the two domains opens approximately 180° by rotation around an ‘elbow’ centered roughly on amino acid 385, and the catalytic site comes to rest in the correct position and orientation to cleave the bottom strand of the DNA, 13 bp away. Using a clever combination of two FokI mutant enzymes, one (D450A) binding-proficient but catalysis-deficient, the other (N13Y) binding-deficient but catalysis-proficient, Steve Halford's group confirmed this strand-specificity experimentally. When FokI binds to its recognition sequence, they report, the catalytic site of the bound molecule cleaves the bottom strand at the ‘distal’ site (+13), and the catalytic site of the recruited molecule cleaves the top strand at the ‘proximal’ site (+9) (403). Whether the same strand-specificity holds true for other Type IIS enzymes remains to be seen.

#### Type IIT

‘Type IIT’ was intended for REases that act as he**t**erodimers, and comprise **t**wo different subunits. Among the original examples, Bpu10I (CC|TNAGC) and BbvCI (CC|TCAGC) fit this description (324,404–405), but BslI (CCNNNNN|NNGG) is now known to be an alpha(2):beta(2) heterotetramer (23), and is better thought of as an unusual Type IIP (**p**alindromic) REase. Type IIT enzymes, today, are perhaps more usefully defined as REases that have **t**wo different *catalytic sites*. Some of these enzymes are heterodimers (e.g. BbvCI; Bpu10I; BtsI, BsrDI and BspD6I (406)). Others are single-chain proteins with two distinct catalytic domains (e.g. Mva1269I (126); BtsCI (407); AciI, BsrI, BssSI and BsrBI). All of these enzymes recognize asymmetric sequences and cleave within, or very close to, only one side of the sequence. In some cases, the two subunits/domains are of similar size, and both participate in sequence recognition as well as in catalysis (e.g. BbvCI; BmgBI). In others, the subunits/domains are of different size, and the larger subunit recognizes the sequence in its entirety and cleaves one strand, while the smaller subunit lacks sequence specificity and just cleaves the second strand (e.g. BsrDI; BtsI; BspD6I). In general, Type IIT REases are accompanied by two separate MTases, one for modifying each strand of their asymmetric recognition sequence. In some systems, these MTases are individual proteins, in others they are joined into a single protein chain. Because Type IIT REases, as defined here, have two different catalytic sites they can be converted into strand-specific nicking endonucleases by mutating one site or the other (127,324), or by eliminating the small subunit (311). See Chan *et al.* for a recent review (408).

#### Grouping by catalytic site

Despite the numerous forms in which they occur, Type II REases are considered to be variations on three catalytic themes, for the most part, termed ‘PD-D/EXK’ (e.g. EcoRI), ‘HNH’ (e.g. KpnI (237)) and ‘GIY-YIG’ (e.g. Eco29kI (370)) for the amino acid motifs that comprise their catalytic sites. These motifs recur in other kinds of nucleases, including homing endonucleases, Holliday-junction resolvases and exonucleases (8). PD-D/EXK endonucleases were described in an earlier section; we discuss the other kinds of catalytic sites here.

#### HNH enzymes

Based on bioinformatics analysis, the next most common class of Type II REases after the PD-D/EXK enzymes are the ‘HNH’ enzymes which include KpnI (GGTAC|C (237)), MboII (GAAGA 8/7), SphI (GCATG|C) and several others (26). Non-specific endonucleases (e.g. the *Serratia* nuclease and colicins), homing endonucleases (e.g. I-PpoI and I-HmuI) and Holliday-junction resolvases also belong to this class (409–411). The catalytic residues of PD-D/EXK enzymes can sometimes be recognized by eye in amino acid sequences, but those of HNH enzymes rarely can since they vary, and are spread out. Sokolowska *et al.* (238) describe the often weak connection that exists between ‘H-N-H’ and the residues that actually form these catalytic sites. The HNH catalytic residues are sometimes embedded in a structure termed a ββαβ fold, in which a zinc ion is coordinated by four cysteine residues in two groups of two (CXXC…CXXC). The occurrence of this motif within a sequence can be indicative of an HNH catalytic site, as it is in Hpy99I (CGWCG|) (238), but this is far from definitive. Many Cys4-Zn^2+^ motifs are not associated with catalytic sites, yet others are associated with PD-D/EXK sites (e.g. DpnI) and variants (e.g. Vsr (412)). In the context of HNH sites, the Zn^2+^ ion is not catalytic but rather acts to maintain the integrity of the fold. And unlike conventional zinc-finger domains that function in DNA sequence recognition, those of HNH REases—there can be more than one in each subunit—perform structural roles unrelated to sequence recognition.

HNH REases require a divalent cation such as Mg^2+^ or Mn^2+^ for catalysis and some, such as MnlI (CCTC 7/6) (413) and HpyAV (CCTTC 6/5) (414), have been reported to use a variety of other ions including Ni^2+^, Co^2+^, Zn^2+^ and even Ca^2+^. Why these serve for catalysis in HNH site (and GIY-YIG sites, below) but not in PD-D/EXK sites remains unclear. Only a single metal ion is present at the catalytic sites in the crystal structures of Hpy99I (Na^+^) and PacI (Ca^2+^), both Type IIP homodimers. It is coordinated in the same way in both, by six oxygen atoms: one each from the side chains of Asp (D) and Asn (N), two from the target phosphate (proS and the 3′-leaving oxygen), and two from water molecules. In Hpy99I and other HNH sites, His (H) is positioned on the 5′-oxygen side of the target phosphate to act as the general base and assist in the creation of the attacking hydroxide ion (415). In PacI (TTAAT|TAA), tyrosine is positioned to be the general base, instead. (22). Despite an unfavorable p*K_a_* of 10, it seems plausible that this tyrosine exists in the phenolate state (-O^−^) before DNA binding, and reverts to the un-ionized state (-OH) by de-protonating the nucleophilic water molecule when close to negatively charged DNA phosphates.

#### GIY-YIG enzymes

A small number of REases including Cfr42I (CCGC|GG), its isoschizomer Eco29kI (372), and Hpy188I (TCN|GA) (240), use a third class of catalytic site termed ‘GIY-YIG’ (26). The DNA co-crystal structures of Eco29KI and Hpy88I, both Type IIP homodimers, have been solved (239). A single Na^+^ ion is present in the Hpy188I catalytic site, coordinated by one amino acid side chain (Asp), three water molecules, and by two phosphate oxygens—proS and the 3′-leaving group. This is same coordination as occurs in the HNH REases discussed above, except that an extra water molecule takes the place of the second amino acid, Asn. Eco29kI crystallized without a metal ion at the catalytic site but the organization is similar. In both structures, the nucleophilic water molecule is positioned and oriented to attack the phosphorus by H-bonds to one main chain carbonyl oxygen and to the side chain oxygen of the tyrosine of the first GIY motif. The latter is presumed to be in the phenolate (-O^−^) state and to act as the general base much as in PacI, with assistance, perhaps, from adjacent residues. Like HNH REases, GIY-YIG REases can use a variety of divalent metals for catalysis in addition to Mg^2+^ and Mn^2+^.

An interesting difference between the HNH and GIY-YIG sites on one hand, and the PD-D/EXK sites on the other, concerns the position of the metal ion. In the former, it contacts two oxygen atoms of the target phosphate group, the proS non-bridging oxygen *and* the 3′ leaving oxygen (238,241). In this position, the metal ion is beyond coordination range of the nucleophilic water, which cannot therefore originate from its hydration sphere. The invariant metal ion of PD-EXK sites, on the other hand, contacts only the non-bridging phosphate oxygen, and is often close enough to coordinate, and help orient, the nucleophilic water. In PD-D/EXK sites in which as second metal ion is present, it often occupies approximately the same position as the single metal ion of the HNH and GIY-YIG sites, and is coordinated in a similar way to both phosphate oxygens. The ability of HNH and GIY-YIG sites to use a variety of metal ions for catalysis while PD-D/EXK sites use only Mg^2+^, and occasionally Mn^2+^, might be related in some way to the different placements and coordinations of the ions.

#### Phospholipase D enzymes

The Type IIS enzyme BfiI (ACTGGG 5-7/4) and its closely related isoschizomer, BmrI, differ strikingly from other REases in both organization and catalysis. They use a metal-independent catalytic site, termed PLD belonging to the Phospholipase D superfamily, and they cleave DNA one strand at a time in an unusual way involving a covalent enzyme–DNA intermediate (309). BfiI acts as a homodimer. The C-terminal half of each subunit forms a DNA-binding domain, which resembles B3-like plant transcription factors (416,417). The dimer binds to two recognition sequences at once (418) but has only one catalytic site, which is located at the interface of the two N-terminal domains, as it is in the EDTA-resistant Nuc endonuclease from *Salmonella typhimurium* (419).

BflI cleaves the bottom strand first (+4) and then, more slowly and with some variability, the top strand. Its catalytic site contains symmetrically disposed His105-X-Lys107 (HXK) residues, typical of PLD enzymes, and additional conserved catalytic residues including N125 and E136. In the first step of the strand-hydrolysis reaction, one of the His residues is proposed to act as the nucleophile, while the other is proposed to act as a general acid to protonate the 3′-O leaving group. This results in the formation of a 3′-OH on one side of the break and a 5′-phospho-histidine covalent intermediate on the other. In the second step, in-line attack by a hydroxide, or some other nucleophile (309), displaces the histidine and generates a 5′-phosphate, which retains its original stereoconfiguration (327). The same catalytic site then transfers to the top DNA strand to hydrolyze that. In principle, since the catalytic site of BfiI is symmetric, it should be able to accommodate the opposite polarity of the top strand by switching the roles of the two histidines and working in reverse, as was originally proposed (326). Surprisingly, BfiI appears not to do this, and instead is reported to swivel the catalytic site by 180° so that the same residues perform the same reaction on both DNA strands (327).

Although BfiI appeared unique when discovered (236), PLD-type REases are far from rare. REBASE BLAST analysis identifies several other isoschizomers of BfiI in addition to BmrI, and over 40 putative enzymes that have the conserved HXK catalytic residues but in all likelihood different organizations and recognition sequences. One cluster of related enzymes includes NgoFVII and AspCNI (GCSGC; cleavage site variable), which have been partially characterized. It is easy to imagine these enzymes binding to their quasi-symmetric recognition sequence as homodimers with a single composite catalytic site. It will be interesting to see whether this catalytic site is bi-directional and can work in reverse, or if these enzymes detach, rotate and reattach in order to hydrolyze both strands, much like the monomeric Type IIP REases such as BcnI (133).

#### ‘Half-pipe’ enzymes

The PabI nuclease (226 aa; GTAC) was identified by bioinformatics analysis of the genome of the archaeon *Pyrococcus abyssi* (420). Its genomic location suggested it might mediate genetic rearrangements, and its proximity to the gene for a companion MTase (421) implied that it was a small, unremarkable, Type IIP REase. PabI was found to cleave DNA in the absence of divalent metal ions, and was reported to leave a 2-base, 3′-overhang: GTA|C. Amino acid sequence analysis revealed little similarity to other REases and, when crystallized without DNA, its structure proved to be unique and was assigned its own fold, termed ‘half-pipe’ (422). The crystal structure of PabI with specific DNA was solved very recently, and shows why this enzyme is so unusual: it is not an endonuclease, after all (423). PabI binds to its symmetric recognition sequence as a homodimer, and flips all four purines out from the helix, leaving the pyrimidines intra-helical, but orphans. And rather than catalyzing phosphodiester bond hydrolysis, PabI is a DNA-adenine glycosylase. It leaves the phophodiester backbone intact, and instead excises both adenine residues to create apurinic sites opposite the thymines. At the high temperature at which *P. abyssi* lives (95°C), strand hydrolysis is thought to proceed spontaneously following this de-purination (423). Close isoschizomers of PabI are ubiquitous in strains of *H. pylori* (e.g. HpyJ99XII), and at the moderate temperature that these organisms live (37°C), strand hydrolysis is thought to be catalyzed by apurinic-apyrimidinic endonuclease (424). Surprisingly, not only does PabI not resemble REases, it does not resemble N-glycosylases, either!

Thousands of Type II REases are known; hundreds have been characterized but most have not. Bioinformatics analysis and structure-guided sequence alignments have allowed approximately two-thirds of these to be assigned to one or other of the three main families, PD-EXK (267,425-426), HNH (427) and GIY-YIG (428). More can be assigned to the PLD-family or the PabI-group, but others cannot be assigned to any family and remain mysteries (26,363). They could be fringe members of the conventional families that have diverged beyond recognition or, like PabI, they could be examples of new, as yet uncharacterized, folds and DNA-degradation mechanisms.

#### Grouping by quarternary structure

Since the substrates of REases are duplex DNA molecules, cleavage requires two catalytic reactions, one for hydrolyzing each DNA strand. Type II REase quarternary organizations can often be understood in terms of the different ways in which two catalytic sites can be brought to act on opposite strands in the vicinity of the same DNA sequence. REases that act as dimers generally possess two catalytic sites; these are identical in homodimers such as HindIII and EcoRI, but different in heterodimers such as BbvCI and BsrDI (324,406). Dimeric REases of both kinds usually cleave both DNA strands in a single binding event. Some monomeric REases, such as HinP1I (130) and MvaI (131), possess only one catalytic site and cleave DNA in two steps, hydrolyzing one strand and then the other immediately afterward. Surprisingly, they do this without detaching from the DNA and returning to bulk solution. Instead, they release the recognition sequence after the first nick, and then randomly slide along the DNA and rotate until the sequence is recaptured in opposite orientation (133). Other monomers, such as BsrI (406) and Mva1269I (127), represent single-chain fusions of ancestral heterodimers. They possess two different catalytic sites within the one polypeptide chain, and generally cleave both DNA strands in one binding event. These enzymes can exhibit marked strand preference, such that one strand must be cleaved by one of the catalytic sites before the second can be cleaved by the other catalytic site (127). Whether this is due to a structural peculiarity of the second catalytic site, or to a biochemical peculiarity in the way it catalyzes the reaction, is not known.

Numerous Type II REases, including many members of the Type IIS subclass, and essentially all members of the Type IIB, C and G groups, possess only one catalytic site and bind to their recognition sequence in an inactive form. Activation is thought to occur by transient dimerization of the catalytic domain with an identical catalytic domain from a second enzyme molecule either bound to another recognition site or, with lesser effect, unbound. Dimerization activates both catalytic sites, and so these enzymes generally cleave both DNA strands at once without the release of nicked intermediates. The need for transient dimerization accounts for the low activity of many REases on substrates with only one recognition site, and explains why activity often increases in the presence of oligos that contain the recognition sequence.

Many restriction enzymes cleave DNA as multimers bound to two recognition sequences at once. Such widespread behavior must confer a significant selective advantage, one that has to do, perhaps, with carefully distinguishing host DNA that must be saved, from foreign DNA that must be destroyed. REases that cleave by transient dimerization automatically monitor two sequences at once when both members of the partnership are bound to DNA specifically. This might be the underlying reason why so many REases operate in this way instead of simply acquiring a second catalytic site, a trivial step in evolutionary terms. REases that bind to their recognition sequences as homodimers already have both catalytic sites needed for cleavage. Some, such as NgoMIV and SfiI, nevertheless monitor two recognition sequences at once by assembling into tetramers of two back-to-back homodimers. Viewed end-on, the two duplexes in these tetramers cross each other at an angle of 60° in an ‘X’ configuration, and both are cleaved at the same time (359).

### Protein engineering of REases—tools for gene targeting

#### REase variants

Soon after the structure of the EcoRI–DNA complex was determined (247), attempts were made to change the specificity of EcoRI by substituting the amino acids involved in base-specific interactions (for example (254)). Substitutions were made according to suggestions a decade earlier that certain amino acids were ideally suited to juxtapose certain bases due to H-bond complementarity. Asparagine and glutamine were ideal for adenine, it was proposed, and arginine was ideal for guanine (91). These particular juxtapositions and several others (429) are common in sequence-specific DNA-binding proteins, we now know, and represent a ‘recognition code’ of sorts, albeit one that is so variable due to alternative amino acids, and H-bonds with atoms of the protein main chain, that it has little predictive power. The substitutions introduced into EcoRI and EcoRV, and subsequently into other REases, usually resulted in a decrease in activity, but without exception failed to produce substantial changes in specificity. The reason for these failures has become clearer with time: recognition is a highly cooperative and redundant process, involving not only amino acids in direct contact with the bases and the backbone, but also structured water molecules and an intricate network of buttressing interactions (306). Even for very well characterized REases, the properties that determine specificity and selectivity are difficult to model with the available structural information (430). Furthermore, the crystal structure of the recognition complex represents a form of the ‘ground state’, but catalysis involves the ‘transition state’, which may depend upon additional interactions not evident in the crystal structure.

In order to change specificity, the functional groups of amino acids must be positioned in three dimensions within the DNA-binding site in precise complementarity with the bases they are to juxtapose. This demands structural accuracy far beyond what can be achieved by gross amino acid substitutions. Notwithstanding, some Type IIG combined RM enzymes have evolved DNA-binding domains with C-alpha structures that allow them to undergo specificity changes naturally at certain base pair positions. Such changes confer a selective advantage because it allows prokaryotes to side-step the resistance to restriction that constantly evolves among its viruses. Almost invariably, these changes in specificity involve switches of two amino acids at once—one for each base of the base pair—and they can be replicated in the laboratory by site-specific mutagenesis to achieve robust changes of specificity. For example, in the MmeI-family of highly homologous RM enzymes that recognize 6–8 bp asymmetric sequences, specificity for GC at certain positions can be routinely changed to CG, and vice versa, by substituting Glu…Arg (E…R) pairs for Lys…Asp (K…D) pairs, and certain other equivalent amino acid combinations (374).

REases normally produce a double-strand cut, but a few can be altered to cut only one strand—to ‘nick’ the DNA, that is (see Chan *et al.* (408) for a recent review). Nicking enzymes are useful for a variety of DNA manipulations, including the preparation of substrates for DNA repair studies (431), the generation of DNA molecules with long overhangs or with gaps, and the creation of 3′-OH termini for labeling, for genomic mapping by optical bar-coding, and for isothermal DNA amplification. Nicking enzymes can be isolated as the principal (large) subunits of some heterodimeric REases (324,406,432–433), or they can be engineered by generating homodimers (434–436) or heterodimers (324) with one active catalytic site and one inactive catalytic site (435). The former enzymes are unusual because their catalytic sites can act alone. BstNBI (GAGTC 4/5±), for example (and the identical BspD6I), comprises two subunits of different size and function (311). One (604 aa) recognizes the DNA and contains the catalytic site for top-strand hydrolysis; the other (186 aa) contains the catalytic site for variable hydrolysis of the bottom strand. In the presence of both the subunits, the DNA is cleaved (432), but in the presence of only the large subunit (‘Nt.BstNBI’), the DNA is efficiently and accurately nicked in only the top strand (433,437). The catalytic sites of most Type IIS enzymes are inactive unless dimerized, whereas the top-strand catalytic site of BstNBI is active either way.

#### The break-through with fusion proteins

**Figure 13.**
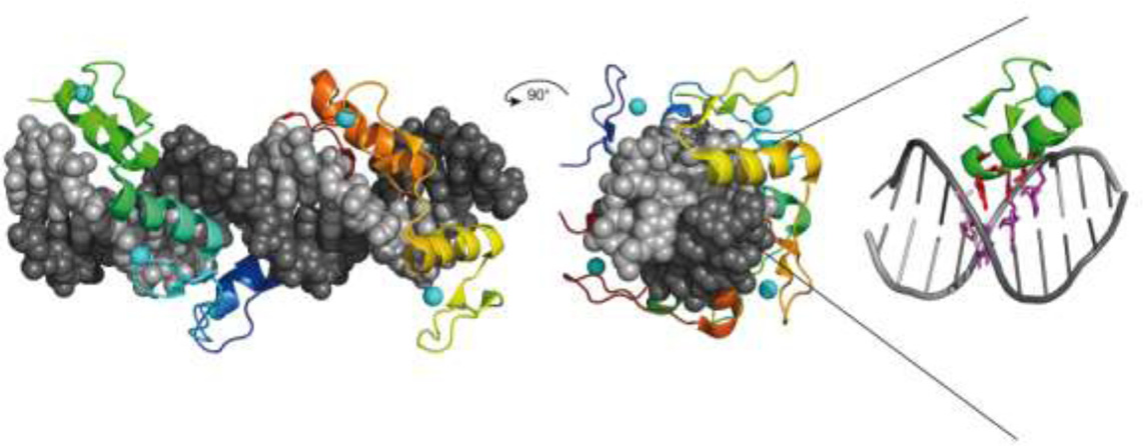
Mode of DNA binding by zinc finger proteins: each finger recognizes approximately three base pairs of the recognition sequence. For one zinc finger the amino acids forming essential base contacts (residues at positions 1, 2, 3, 6 of each helix) are shown in purple.

Type II REases are among the most specific enzymes known. On average, they recognize and cleave one site every 1 × 4*^n^* base pairs for symmetric sequences, and 2 × 4*^n^* base pairs for asymmetric sequences, where *n* is the length of the recognition sequence, typically 4–8. For precise gene targeting in the complex genomes of eukaryotes, only a single cut at a defined location is desirable. Achieving this degree of specificity requires a recognition sequence of about 20 bp in length. To accomplish this, Srinivasan Chandrasegaran at Johns Hopkins School of Medicine pioneered a new approach of ‘modular design and assembly’ (438). Analysis of the Type IIS REase, FokI (GGATG 9/13), showed that the enzyme was organized in a different way than EcoRI and EcoRV. Whereas the catalytic and recognition residues of the latter are integrated into a single protein domain, in FokI they are separate. FokI has an N-terminal DNA-binding domain (BD), and a C-terminal, non-specific, cleavage domain (CD) that hydrolyzes DNA outside of the recognition sequence roughly one turn of the helix away (389,439-442). Changdrasegaran's group constructed novel fusion proteins consisting of DNA-binding modules from eukaryotic proteins joined to the FoKI CD module (388). Their most successful fusion used the DNA-binding domain from a zinc finger protein joined to the FokI CD to create what is now termed a ‘zinc finger nuclease’ (ZFN) (438). ZFNs typically contain a series of three to six zinc fingers. Each zinc finger comprises ∼30 aa that fold into a characteristic ββα structure that coordinates one Zn^2+^ ion via two cysteine and two histidine residues (443,444). Each zinc finger recognizes a three base pair target sequence through four contact amino acids that project from the α helix into the major DNA groove (445,446) (Figure 13).

The use of zinc fingers as specific DNA-binding modules offers the advantage that they are ‘programmable’. The specificities of individual fingers can be changed to some extent by mutagenesis, and the order of the fingers in an array can be changed at will by gene synthesis. In principle, almost any sequence in a complex genome can be targeted with a carefully selected zinc finger array, although in practice this is easier said than done. The non-specific FokI cleavage domain of ZFNs does not contribute to specificity, but it has a property that greatly enhances the accuracy and utility of ZFNs. On its own, the FokI CD is inactive. In order to cleave DNA, two CDs from oppositely oriented molecules must dimerize transiently (329). Positioning two CDs close together on DNA increases the likelihood that this will occur. In FokI-based ZFNs, two separate zinc finger arrays are designed to bind to adjacent sites in the DNA in opposite orientations. With two different three-finger ZFNs, a 2 × (3+3+3) = 18-bp sequence that is unique in the human genome can be recognized and cleaved (447). Pairs of ZFNs have been used with considerable success in this way for gene targeting (448–450), although evidence is mounting that they are not as specific as might be expected (451,452), and that cleavage at unintended sites also occurs. Part of this ‘off-target’ cleavage is due to homodimer formation, and can be reduced by mutating the amino acids of the CD dimerization surface (453,454). Part might also be due, as pointed out by Halford *et al.*, to dimerization between a specifically bound ZFN and one that is not specifically bound. The FokI CD is inherently compromised, they suggest, because its dimerization mechanism does not preclude off-site targeting (395). Recently, a novel zinc-finger nuclease platform was described using a derivative of PvuII as a sequence-specific catalytic domain instead of the FokI CD. PvuII adds an extra element of specificity when combined with zinc fingers, and ZF-PvuII nucleases are designed such that a PvuII site (CAG|CTG) occurs naturally between the two ZF-binding sites. In contrast to the ‘analogous’ ZF-FokI nucleases, neither excess enzyme over substrate nor prolonged incubation times results in off-target cleavage by ZF-PvuII nuclease pairs *in vitro* (455).

**Figure 14.**
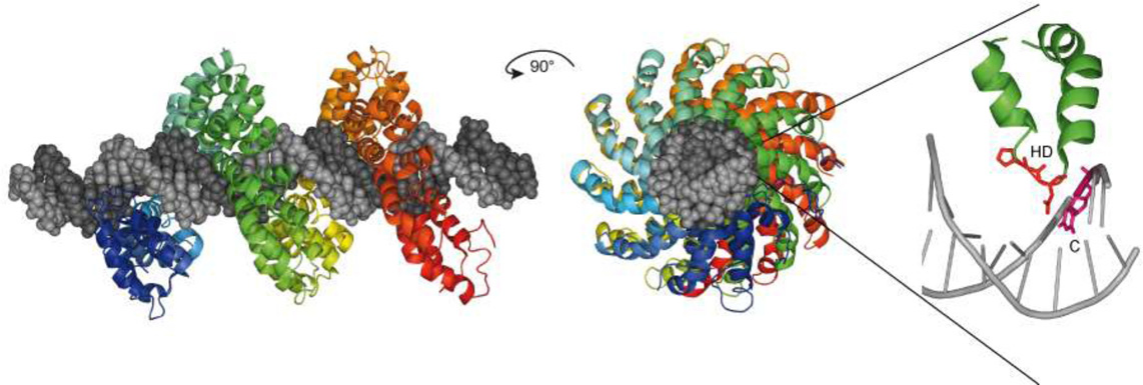
Mode of DNA binding by a TAL effector: DNA binding is mediated by a central region comprising a series of near-identical tandem repeats, usually 34 residues in length. The amino acid residues at positions 12 and 13 are hyper-variable (the ‘repeat variable di-residue’, or RVD). The side chain of residue 13 of each repeat determines the base recognized by that repeat in a simple but ambiguous 1:1 ‘recognition code’. The repeats precisely track the sense strand of the DNA, and so the order of the repeats determines the bp sequence recognized. The RVD (HD) is shown in red.

The design and selection of zinc finger arrays to make pairs of ZFNs for gene targeting is complex and costly. After the DNA-binding domains of transcription activator-like effector (TALE) proteins were shown to be modular, and to recognize DNA in a simple 1 module:1 base fashion (456,457), they began to be used instead of ZF arrays to engineer programmable nucleases for gene targeting. Natural TALE proteins contain a variable number (up to 35) of nearly identical, ∼34 aa tandem repeats. The amino acid at position 13 in each repeat (the second residue of the ‘repeat-variable di-residue’ or RVD) is responsible for base-recognition. The repeat arrays form a right-handed super-helix that spirals around the DNA with astonishing elegance, following the track of the major groove for several turns. The individual repeats are left-handed two-helix bundles that, one after the other, juxtapose aa 13 of each RVD to adjacent bases in the sense strand of the DNA (458–460), (Figure 14).

Because of their simple design, predictable sequence specificity and ease of synthesis, TALE-based nucleases (TALENs) have largely replaced ZFNs as the tools of choice for genome engineering. So far, TALEs have been used mainly in conjunction with the FokI CD (461,462). It remains to be seen whether TALENs are less prone to off-target cleavage than ZFNs (463) and whether alternative DNA-cleavage modules from other Type II REases can offer advantages over the cleavage domain of FokI (464,465). Gene targeting requires precisely positioned incisions in genomic DNA in order to stimulate repair by homology-directed genetic recombination (HR). It has been argued that it might be better to cut only one DNA strand for this purpose, using a nicking domain rather than a cleavage domain, as this would decrease competing repair by error-prone, non-homologous end joining (NHEJ) (408). Such engineered nickases have been used in conjunction with zinc fingers (466–468), TALE proteins (465) and methyl CpG binding domains (469), and are proving to be very effective.

### Epilogue

Type II REases have come of age. In doing so, they have changed the landscape of molecular biology in ways barely imaginable a few decades ago. It all started with the observation that phage sometimes infect new bacteria very poorly. What might have been dismissed as just a nuisance was studied instead and grew into the microbial field of ‘host-controlled restriction and modification’, an academic curiosity of little broader significance. But as its underlying biochemistry emerged, in the hands of a few skilled scientists, the use of ‘restriction enzymes’ as laboratory tools for DNA analysis and experimentation began to be considered. As Louis Pasteur said in a lecture delivered at the University of Lille (7 December 1854), ‘*Dans les champs de l'observation le hasard ne favorise que les esprits prepares*’ (‘In the fields of observation chance favors only the prepared mind’). Fueled by the subsequent discovery of the Type II enzymes in the early 1970s, and by the inventions of gene cloning and mapping, a revolutionary new technology—‘Recombinant DNA’—sprang into being. This technology has since transformed the life sciences and medicine, and has seeded a multitude of enterprises, large and small (43). To Type II REases we owe many billions of dollars of economic activity, thousands of jobs and careers, and staggering advances in knowledge and understanding. Few examples as this speak so clearly of the importance to society of investments in unencumbered, curiosity-driven, basic research. To quote Pasteur once more ‘*Il n'existe pas de catégorie de science qui puisse être désignée comme étant appliquée’. Il y a la science et les applications de celle-ci, réunies comme le sont le fruit et l'arbre qui le porte*’ (1871). (‘There does not exist a category of science to which one can give the name applied science. There is science and its applications, bound together as the fruit of the tree that bears it’.)

## SUPPLEMENTARY DATA

Supplementary Data are available at NAR Online.

SUPPLEMENTARY DATA
