# Supplementary material for: Type II restriction endonucleases—a historical perspective and more
Source: Nucleic Acids Res. 2014 Jun 26;42(12):7489–527. doi: 10.1093/nar/gku447 (PMC4081073; doi:10.1093/nar/gku447)
Supplement: SUPPLEMENTARY DATA [file supp_42_12_7489__index.html]

Type II restriction endonucleases—a historical perspective and more — Type II restriction endonucleases—a historical perspective and more — SUPPLEMENTARY DATA 

# Type II restriction endonucleases—a historical perspective and more

## SUPPLEMENTARY DATA

**Files in this Data Supplement:**

- SUPPLEMENTARY DATA
