# Supplementary material for: Type II restriction endonucleases—a historical perspective and more
Source: Nucleic Acids Res. 2014 Jun 26;42(12):7489–527. doi: 10.1093/nar/gku447 (PMC4081073; doi:10.1093/nar/gku447)
Supplement: SUPPLEMENTARY DATA [file supp_gku447_Pingoud_et_al_SUPPLEMENTARY_MATERIAL.pdf]

SUPPLEMENTARY FIGURE & TABLE LEGENDS

Supplementary Figure S1. Procedures used for cloning R-M systems. Plasmid libraries are prepared and digested with the restriction enzyme whose gene is sought (middle flow diagram). The digests are used to transform *E.coli*, and survivors are screened individually, or pooled and cycled through a second round of digestion. This 'methylase selection' yields complete R-M systems sometimes, but more often only the M gene. The libraries can be exposed to phage to select for cells that carry the complete R-M system and are able to restrict, but this method is unreliable. When only the M gene is isolated, adjacent fragments can be identified and analyzed and the R gene recovered non-selectively. N-terminal amino acid analysis of the purified REase, and internal tryptic peptide analysis, are helpful in these cases (right flow diagram). Increasingly, R-M systems are now identified by whole-genome sequencing using 454- and PacBio-machines and bioinformatics analysis (left flow diagram). The systems are then retrieved by PCR or gene synthesis. PacBio is particularly useful in this regard because it delivers the gene sequences, and also often the recognition sequence and the position of DNA modification (1). Cloning R-M systems has brought two major benefits: better, cheaper, laboratory reagents for DNA manipulation, and extensive insight into the organizations, evolution and biochemistry of the proteins involved.

Supplementary Table 1. R-M systems cloned at New England Biolabs, Inc., 1981-2013. The list is not exhaustive, and Type IV systems are omitted. The table is organized according to the research group responsible for the cloning. Some projects involved more than one group resulting in duplicate table entries. Several entries repeat published work reported by others. Some projects spanned several years; the date generally refers to the cloning of the REase gene. Some dates are estimates. The sequences of most of the systems are available in REBASE and GenBank, although many have not been formally reported in the scientific literature. **Group A:** Geoff Wilson, Donald Nwankwo, Chuck Card, Rebecca Croft, Keith Lunnen, Tineka Jager, Elizabeth Van Cott, Russ Camp, Shu-Zi Chen, Janet Barsomian, Mary Looney, Barton Slatko, Laurie Moran, Bo-Hong Zhang, Marta Meda, Dan Heiter, Jonathan O'Driscoll, Hua Wang. **Group B:** Rich Roberts, Iain Murray, Alexy Fomenkov, Rick Morgan, Brian Anton, Juliane Krebes. **Group C:** Joan Brooks, Kim Howard, Karen Silber, Laura Sznyter, Peter Nathan, Brian Anton. **Group D:** Shuang-yong Xu, Jian-Ping Xiao, Jim Samuelson, Alexey Fomenkov, Zhenyu Zhu, Siu-Hong Chan. **Group E:** Rick Morgan, Carol Polisson, Yvette Luyten. **Group F:** Zhenyu Zhu, Shengxi Guan, Aine Quimby, Janine Borgaro. '454' and 'Pac' refer to systems identified by bioinformatics analysis of 454 and Pac Bio genome sequencing projects under the direction of group B. 'CSH' refers to clones isolated or analyzed at Cold Spring Harbor Laboratory (2,3).

Supplementary Table S2. Restriction enzyme-DNA co-crystal structures solved by X-ray crystallography. Enzymes crystallized without DNA are omitted. Column 1: year the structure was published. Column 2: name of the enzyme. Column 3: the DNA sequence recognized and the position of cleavage ('|'). PabI (last entry) acts as a sequence-specific DNA-adenine N-glycosylase rather than an endonuclease; catalysis creates apurinic sites that are thought to undergo phosphodiester bond hydrolysis spontaneously (*). Most recognition sequences are symmetric and cleavage occurs at the same position on the complementary strand. Column 4:

the oligomeric form in which the enzyme cleaves DNA. ‘1’: binds to recognition sequence and cleaves as a monomer; ‘1-2’: binds as monomer but dimerizes transiently to accomplish cleavage; ‘2’: binds and cleaves as a homodimer; ‘2-4’: binds as a homodimer, but cleaves as a dimer of dimers. Column 5: publication reporting the structure.

Supplementary Table S3. Approximate numbers of Type II REases in each subtype as described in the text. Adapted from REBASE (May 24, 2014). Subtypes P, A, C, G, S, and M include both characterized enzymes, and putative enzymes identified by bioinformatics analysis of sequenced genomes and meta-genomic samples. Some of the subtype M enzymes included here might better be classified as Type IV REases. Subtypes H, T, B, E, and F include only characterized REases. Among these, subtypes B, E and F are probably far more common than the numbers shown here suggest.

Supplementary Figure S1. R-M system cloning workflow

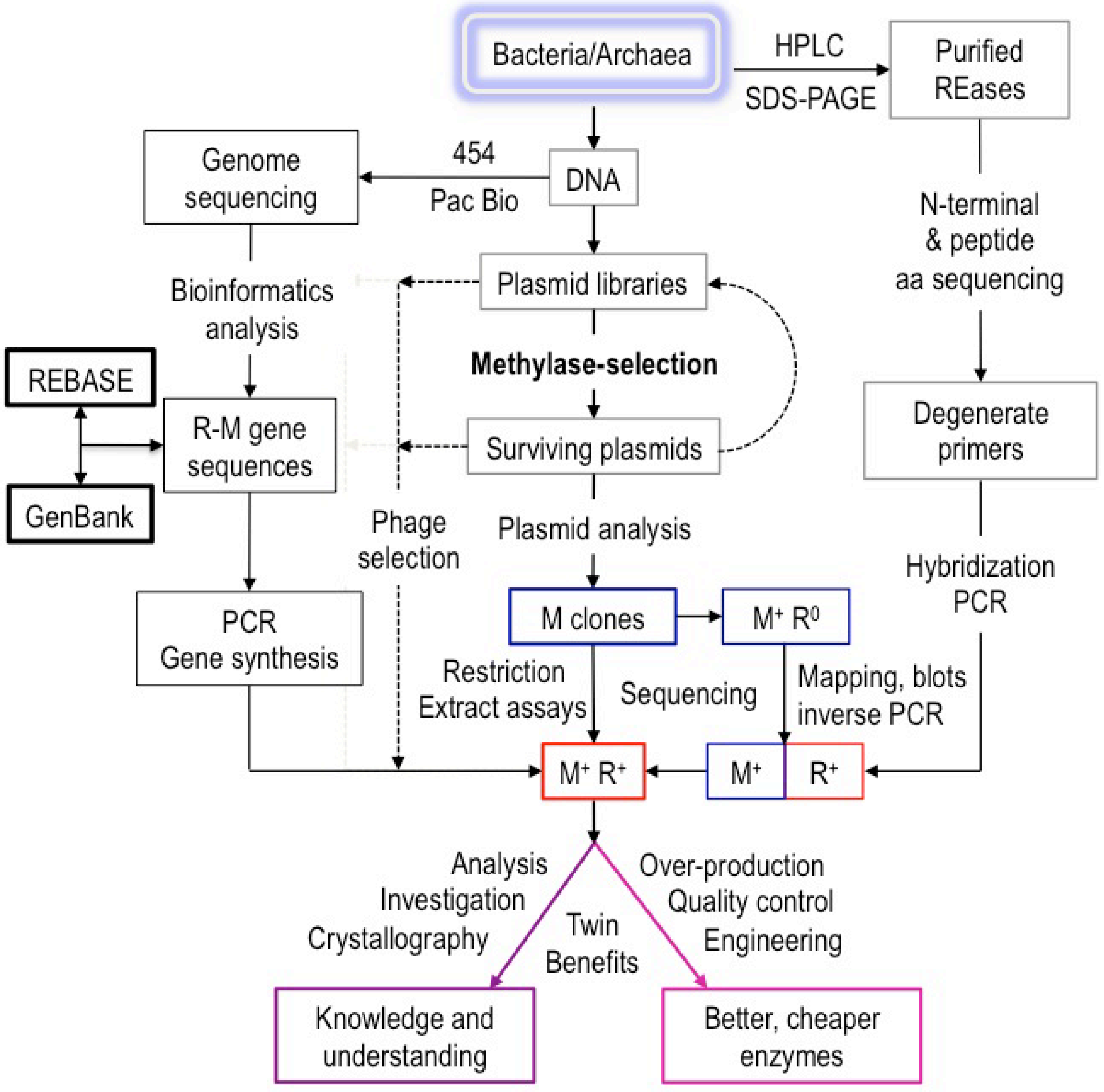

Supplementary Table S1. R-M systems cloned at New England Biolabs, Inc.

Group A

Enzyme	Recognition Sequence	Year
PstI	CTGCAG	1981
PstII	CATCAG 25/27	1981
EcoRI	GAATTC	1982
FokI	GGATG 9/13	1983
HaeII	RGCGCY	1983
HaeIII	GGCC	1983
HhaI	GCGC	1983
HhaII	GANTC	1983
HinfI	GANTC	1983
MspI	CCGG	1983
BamHI	GGATCC	1984
BanI	GGYRCC	1984
DdeI	CTNAG	1984
HgaI	GACGC	1984
HgiAI	GWGCWC	1984
HindII	GTYRAC	1984
HindIII	AAGCTT	1984
TaqI	TCGA	1984
AluI	AGCT	1985
BanII	GRGCYC	1985
BglI	GCC N5 GGC	1985
FnuDII	CGCG	1985
FnuDIII	GCGC	1985
HpaII	CCGG	1985
NlaIII	CATG	1985
NlaIV	GGNNCC	1985
SalI	GTCGAC	1985
AvaI	CYCGRG	1986
AvaII	GGWCC	1986
FnuDI	GGCC	1986
AatII	GACGTC	1987
AccI	GTMKAC	1987
Afl II	CTTAAG	1987
HinP1I	GCGC	1987
NaeI	GCCGGC	1987
NcoI	CCATGG	1987
XbaI	TCTAGA	1987
AvrI	CYCGRG	1988
AvrII	CCTAGG	1988
BalI	TGGCCA	1988
BstYI	RGATCY	1988
MwoI	GC N7 GC	1988
SacII	CCGCGG	1988
Sau96I	GGNCC	1988
SmaI	CCCGGG	1988
SphI	GCATGC	1988
XmaI	CCCGGG	1988
XmaIII	CGGCCG	1988

Enzyme	Recognition Sequence	Year	Note
Afl III	ACRYGT	1989	
BbvI	GCAGC 8/12	1989	
NciI	CCSGG	1989	
SfiI	GGCC N5 GGCC	1989	
AmeI	GTGCAC	1990	
AmeII	GCCGGC	1990	
BfaI	CTAG	1990	
EarI	CTCTTC 1/4	1990	
AciI	CCGC	1991	
ApoI	RAATTY	1991	
AscI	GGCGCGCC	1991	
SfaNI	GCATC 5/9	1992	
Tsp509I	AATT	1992	
KasI	GGCGCC	1992	
MscI	TGGCCA	1993	
AgeI	ACCGGT	1994	
AhdI	GAC N5 GTC	1994	
BsrI	ACTGG 1/-1	1994	
RsaI	GTAC	1994	
SnaBI	TACGTA	1994	
BlpI	GCTNAGC	1995	
BsrBI	CCGCTC	1997	
EaeI	YGGCCR	1997	
AclI	AACGTT	1998	
Bsu36I	CCTNAGG	1999	
AlwI	GGATC 4/5	1999	
BbvCI	CCTCAGC	2000	
BssSI	CACGAG	2000	
Cac8I	GCNNGC	2000	
SfoI	GGCGCC	2000	
DrdI	GAC N6 GTC	2001	
MluCI	AATT	2001	
SbfI	CCTGCAGG	2002	
BmrI	ACTGGG 5/4	2002	
CspCI	10/12 CCAC N5 TTG 12/10	2003	
Acc65I	GGTACC	2004	
BssIMI	GGGTC -3/0	2004	
AleI	CAC N4 GTG	2005	
AfeI	AGCGCT	2006	454
BstNBI	GAGTC 4/4	2006	
BspDI	ATCGAT	2007	454
BssKI	CCNGG	2007	454
BstXI	CCA N6 TGG	2007	
NcuI	GAAGA 8/7	2007	454
NgoAVIII	10/12 TCA N5 GTC 13/11	2007	
SdeOSI	10/12 TCAY N4 GTC 13/11	2007	
BtgZI	GCGATG 10/14	2008	454
PvuI	CGATCG	2008	

Group B

Enzyme	Recognition Sequence	Year	Note
BsuRI	GGCC	1985	CSH
MspI	CCGG	1989	CSH
TspMI	CCCGGG	2007	
BsaHI	GRCGYC	2008	
Hpy188I	TCNGA	2000	
AbaCI	CTATCAV	2010	
DrdVI	GCAGCC	2011	
CjeFIII	GCAAGG	2012	Pac
CjeFV	GGRCA	2012	Pac
CjeNIII	GKAAYG 19/17	2012	Pac
Hpy99XIII	GCCTA	2013	Pac
Hpy99XIV	GGWTAA	2013	Pac
HpyAXIV	GCGTA	2013	Pac
HpyAXVIII	ACA N8 TAG	2013	Pac
Mba11I	AGGCGA	2013	Pac
Nal45188II	ACCAGC	2013	Pac
Plu4II	CGTARC	2013	Pac
SstE37I	CGAAGAC 20/18	2013	Pac
Cal14237I	GGTTAG	2014	Pac
DvuIII	CACNCAC	2014	Pac
Hpy99XIII	GCCTA	2014	Pac
PaePA71	GAGGAC	2014	Pac

Group C

Enzyme	Recognition Sequence	Year	
DdeI	CTNAG	1985	
BamHI	GGATCC	1986	
BglII	AGATCT		
SphI	GCATGC		
EagI	CGGCCG		
SacII	CCGCGG		
XhoI	CTCGAG		
XhoII	RGATCY		

Group D

Enzyme	Recognition Sequence	Year	
AatII	GACGTC	1992	
EcoO109I	RGGNCCY	1992	
BsoBI	CYCGRG	1993	
TfiI	GAWTC	1993	
Tth111I	GAC N3 GTC	1993	
ApaLI	GTGCAC	1994	
MfeI	CAATTG	1994	
SacI	GAGCTC	1994	
ScaI	AGTACT	1994	
SapI	GCTCTTC 1/4	1995	
BssHII	GCGCGC	1996	
BslI	CC N7 GG	1996	
BspHI	TCATGA	1996	
TseI	GCWGC	1996	
Tsp45I	GTSAC	1996	
AgeI	ACCGGT	1997	
NspI	RCATGY	1997	
BsrFI	RCCGGY	1998	
NheI	GCTAGC	1998	
NspHI	RCATGY	1998	
OkrAI	GGATCC	1998	
BpmI	CTGGAG 16/14	1999	
BsaJI	CCNNGG	2000	
BsmI	GAATGC 1/-2	2000	
BstYI	RGATCY	2000	
TspRI	CASTG	2000	
BsmBI	CGTCTC 1/5	2000	
BseRI	GAGGAG 10/8	2000	
BsaI	GGTCTC 1/5	2001	
PpuMI	RGGWCCY	2001	
BsaWI	WCCGGW	2001	
BsmAI	GTCTC 1/5	2001	
MspAI1	CMGCKG	2001	
AcuI	CTGAAG 16/14	2002	

Enzyme	Recognition Sequence	Year	Note
BsrGI	TGTACA	2002	
BtsI	GCAGTG 2/0	2003	
BtsCI	GGATG 2/0	2003	
N.CviPII	CCD	2003	
PhoI	GGCC	2003	
TmaI	CGCG	2003	
ApeKI	GCWGC	2004	
BpuSI	GGGAC 10/14	2004	
BsmFI	GGGAC 10/14	2004	
CviAII	CATG	2004	
CviKI-1	RGCY	2004	
CviQI	GTAC	2004	
Eco53KI	GAGCTC	2004	
AsiSI	GCGATCGC	2005	
BsrDI	GCAATG 2/0	2005	
EcoNI	CCT N5 AGG	2005	
N.CviQII	RAG	2005	
NarI	GGCGCC	2005	
NruI	TCGCGA	2005	
StuI	AGGCCT	2005	
Tth111II	CAARCA 11/9	2005	
BmrI	ACTGGG	2006	
BspQI	GCTCTTC 1/4	2006	454
BceSI	CGAAG 25/27	2008	
BceSIII	ACGGC 12/14	2008	
BcoDI	GTCTC	2008	
CatHI	CTCTTC 1/4	2009	
HpyAV	CCTTC 6/5	2009	
SauUSI	SCNGS	2009	
N.φGamma	ACCGR	2011	
N.BceSVIII	AYSS	2011	
BsaXI	9/12 AC N5 CTCC 10/7	2012	
BisI	Gm5CNGC	2012	
FspCI	ACCTGC 4/8	2013	

Group E

Enzyme	Recognition Sequence	Year
NlaI	GGCC	1989
NlaIII	CATG	1989
NlaIV	GGNNCC	1989
NotI	GCGGCCGC	1992
BspMI	ACCTGC 4/8	1993
FseI	GGCCGGCC	1994
RsrII	CGGWCCG	1994
MseI	TTAA	1996
BccI	CCATC 4/5	1995
Fnu4HI	GCNGC	1995
MboI	GATC	1995
MboII	GAAGA 8/7	1995
PshAI	GAC N4 GTC	1995
PacI	TTAATTAA	1996
PmeI	GTTTAAAC	1996
SpeI	ACTAGT	1996
PspGI	CCWGG	1997
AseI	ATTAAT	1998
AseII	CCSGG	1998
Hpy99I	CGWCG	1999
Hpy188I	TCNGA	1999
Hpy188III	TCNNGA	1999
HpyCH4III	ACNGT	1999
HpyCH4IV	ACGT	1999
HpyCH4V	TGCA	1999
MjaI	CTAG	1999
MjaIII	GATC	1999
MjaIV	GTNNAC	1999
MjaV	GTAC	1999
EsaBC3I	TCGA	2000
AspCNI	GCSGC	2001
BspCNI	CTCAG 9/7	2001
BstEII	GGTNACC	2002
BseYI	CCAGC	2005
FatI	CATG	2006

Enzyme	Recognition Sequence	Year	Note
	Type IIG RM Enzymes		
MmeI	TCCRAC 20/18	2001	
CstMI	AAGGAG 20/18	2003	
DraRI	CAAGNAC 20/18	2006	
EsaSSI	GACCAC 20/18	2006	
NlaCI	CATCAC 19/17	2006	
NmeAIII	GCCGAG 21/19	2006	
PspOMII	CGCCCAR 20/18	2006	
SdeAI	CAGRAG 21/19	2006	
SpoDI	GCGGRAG 20/18	2006	
ApyPI	ATCGAC 20/18	2007	
CdpI	GCGGAG 20/18	2007	
DrdIV	TACGAC 20/18	2007	
PspPRI	CCYCAG 21/19	2007	
MaqI	CRTTGAC 20/18	2008	
NhaXI	CAAGRAG 20/18	2008	
PlaDI	CATCAG 21/19	2008	
RpaB5I	CGRGGAC 20/18	2008	
AquII	GCCGNAC 20/18	2009	
AquIII	GAGGAG 20/18	2009	
AquIV	GRGGAAG 20/18	2009	
AteTI	GGGRAG	2009	
CcoMI	CAGCAG	2009	
GauT27I	CGCGCAGG	2009	
HauII	TGGCCA 11/9	2009	
Mae7806I	AAGGAG	2009	
MchCM4I	GAGGAG 20/18	2009	
PliMI	CGCCGAC	2009	
RceI	CATCGAC 20/18	2009	
CchII 2010	GGARGA 11/9	2010	
CchIII	CCCAAG 20/18	2010	
RdeGBI	CCGCAG	2010	
RdeGBII	ACCCAG 20/18	2010	
RlaII	ACACAG	2010	
RpaI	GTYGGAG 11/9	2010	
RpaBI	CCCGCAG 20/18	2010	
RpaTI	GRTGGAG 20/18	2010	
Sno506I	GGCCGAG	2010	
DprMRI	GCCCCAG	2011	
NflHI	GCGGAG	2011	
RflFIII	CGCCAG	2011	
RmuAI	CCCGAC	2011	
WviI	CACRAG	2011	
Cgl13032I	GGCGCA	2012	

Group F

Enzyme	Recognition Sequence	Year	Note
AlwNI	CAG N3 CTG	2006	454
BmtI	GCTAGC	2006	454
Bpu10I	CCTNAGC	2006	
BsaHI	GRCGYC	2006	
Hpy166II	GTNNAC	2006	
MslI	CAY N4 RTG	2006	454
PciI	ACATGT	2006	454
PspXI	VCTCGAGB	2006	454
ZraI	GACGTC	2006	454
Bme1580I	GKGCMC	2007	
BciVI	GGATAC 6/5	2007	
BspKT6I	GATC	2007	
BstBI	TTCGAA	2007	
BstZ17I	GTATAC	2007	
FauI	CCCGC 4/6	2007	
NarI	GGCGCC	2007	454
PspOMI	GGGCCC	2007	454
PsiI	TTATAA	2007	
SmlI	CTYRAG	2007	454
BscAI	GCATC 4/6	2008	
BsiHKAI	GWGCWC	2008	
BsrBI	CCGCTC	2008	
BmgBI	CACGTC	2009	454
Bsp1286I	GDGCHC	2009	
BspUI	GCSGC	2009	454
BstAPI	GCA N5 TGC	2009	454
BstNI	CCWGG	2009	454
PflFI	GAC N3 GTC	2009	454
TseI	GCWGC	2009	
BaeI	10/15 AC N4 GTAYC 12/7	2011	454
BaeGI	GKGCMC	2011	
BbsI	GAAGAC 2/6	2011	454
BsaBI	GAT N4 ATC	2011	454
BsiEI	CGRYCG	2011	
EciI	GGCGGA 11/9	2011	454
BsgI	GTGCAG 16/14	2012	
Cac8I	GCNNGC	2012	
DrdI	GAC N6 GTC	2013	454

Supplementary Table S2. Restriction Enzyme-DNA Co-Crystal Structures

Year	Enzyme	Recognition sequence	Active form	Reference
1986; 1990	EcoRI	G|AATTC	2	(4),(5)
1993	EcoRV	GAT|ATC	2	(6)
1994	PvuII	CAG|CTG	2	(7)
1995	BamHI	G|GATCC	2	(8)
1997	FokI	GGATG 9/13	1-2	(9)
1998	BglI	GCCNNNN|NGGC	2	(10)
1999	MunI	C|AATTG	2	(11)
2000	BglII	A|GATCT	2	(12)
2000	NaeI	GCC|GGC	2	(13)
2000	NgoMIV	G|CCGGC	2-4	(14)
2001	BsoBI	C|YCGRG	2	(15)
2004	HincII	GTY|RAC	2	(16)
2004	BstYI	R|GATCY	2	(17)
2005	EcoO109I	RG|GNCCY	2	(18)
2005	HinP1I	G|CGC	1	(19)
2005	MspI	C|CGG	1	(20)
2005	SfiI	GGCCNNNN|NGGCC	2-4	(21)
2006	Ecl18kI	|CCNGG	2-4	(22)
2007	BcnI	CC|SGG	1	(23)
2007	MvaI	CC|WGG	1	(24)
2008	BpuJI	CCCGT	1-2	(25)
2008	NotI	GC|GGCCGC	2	(26)
2008	PspGI	|CCWGG	2	(27)
2008	SgrAI	CR|CCGGYG	2-4+	(28)
2009	EcoRII	|CCWGG	2	(29)
2009	HindIII	A|AGCTT	2	(30)
2009	Hpy99I	CGWCG|	2	(31)
2010	Eco29KI	CCGC|GG	2	(32)
2010	PacI	TTAAT|TAA	2	(33)
2011	Hpy188I	TCN|GA	2	(34)
2011	OkrAI	G|GATCC	2	(35)
2011	ThaI	CG|CG	2	(36)
2012	Bse634I	R|CCGGY	2- 4	(37)
2012	DpnI	Gm6A|TC	1	(38)
2014	BfiI	ACTGGG 5/4	2	(39)
2014	PabI	GTA*C	2	(40)

Supplementary Table S3. Occurrences of Type II subtypes

Type II Subtype	Definition from Roberts et al. 2003 [37]<br>Not all subtypes are mutually exclusive	No. of enzymes listet in REBASE
**P**	Symmetric target and cleavage sites	8697
**A**	Asymmetric recognition sequence	6230
**C**	Symmetric or asymmetric target. R and M functions in one polypeptide	4960
**G**	Symmetric or asymmetric target. Affected by AdoMet	4954
**S**	Asymmetric target and cleavage sites	1221
**M**	Subtype IIP or IIA. Require methylated target	257
**H**	Symmetric or asymmetric target. Similar to Type I gene structure	~ 50
**T**	Symmetric or asymmetric target. R genes are heterodimers	25
**B**	Cleaves both sides of target on both strands	16
**E**	Two targets; one cleaved, one an effector	12
**F**	Two targets, both cleaved coordinately	8

REFERENCES

1. Murray, I.A., Clark, T.A., Morgan, R.D., Boitano, M., Anton, B.P., Luong, K., Fomenkov, A., Turner, S.W., Korlach, J. and Roberts, R.J. (2012) The methylomes of six bacteria. *Nucleic Acids Res.*, **40**, 11450-11462.
2. Kiss, A., Posfai, G., Keller, C.C., Venetianer, P. and Roberts, R.J. (1985) Nucleotide sequence of the BsuRI restriction-modification system. *Nucleic Acids Res.*, **13**, 6403-6420.
3. Lin, P.M., Lee, C.H. and Roberts, R.J. (1989) Cloning and characterization of the genes encoding the MspI restriction modification system. *Nucleic Acids Res.*, **17**, 3001-3011.
4. McClarin, J.A., Frederick, C.A., Wang, B.C., Greene, P., Boyer, H.W., Grable, J. and Rosenberg, J.M. (1986) Structure of the DNA-EcoRI endonuclease recognition complex at 3 angstrom resolution. *Science*, **234**, 1526-1541.
5. Kim, Y., Grable, J.C., Love, R., Greene, P.J. and Rosenberg, J.M. (1990) Refinement of EcoRI endonuclease crystal structure: A revised protein chain tracing *Science*, **249**, 1307-1309.
6. Winkler, F.K., Banner, D.W., Oefner, C., Tsernoglou, D., Brown, R.S., Heathman, S.P., Bryan, R.K., Martin, P.D., Petratos, K. and Wilson, K.S. (1993) The crystal structure of EcoRV endonuclease and of its complexes with cognate and non-cognate DNA fragments. *EMBO J.*, **12**, 1781-17945.
7. Cheng, X., Balendiran, K., Schildkraut, I. and Anderson, J.E. (1994) Structure of PvuII endonuclease with cognate DNA. *EMBO J.*, **13**, 3927-3935.
8. Newman, M., Strzelecka, T., Dorner, L.F., Schildkraut, I. and Aggarwal, A.K. (1995) Structure of BamHI endonuclease bound to DNA: partial folding and unfolding on DNA binding. *Science*, **269**, 656-663.
9. Wah, D.A., Hirsch, J.A., Dorner, L.F., Schildkraut, I. and Aggarwal, A.K. (1997) Structure of the multimodular endonuclease FokI bound to DNA. *Nature*, **388**, 97-100.
10. Newman, M., Lunnen, K., Wilson, G., Greci, J., Schildkraut, I. and Phillips, S.E.V. (1998) Crystal structure of restriction endonuclease BglI bound to its interrupted DNA recognition sequence. *EMBO J.*, **17**, 5466-5476.
11. Deibert, M., Grazulis, S., Janulaitis, A., Siksnys, V. and Huber, R. (1999) Crystal structure of MunI restriction endonuclease in complex with cognate DNA at 1.7 A resolution. *EMBO J.*, **18**, 5805-5816.
12. Lukacs, C.M., Kucera, R., Schildkraut, I. and Aggarwal, A.K. (2000) Understanding the immutability of restriction enzymes: crystal structure of BglII and its DNA substrate at 1.5A resolution. *Nat. Struct. Biol.*, **7**, 134-140.
13. Huai, Q., Colandene, J.D., Chen, Y., Luo, F., Zhao, Y., Topal, M.D. and Ke, H. (2000) Crystal structure of NaeI - an evolutionary bridge between DNA endonuclease and topoisomerase. *EMBO J.*, **19**, 3110-3118.
14. Deibert, M., Grazulis, S., Sasnauskas, G., Siksnys, V. and Huber, R. (2000) Structure of the tetrameric restriction endonuclease NgoMIV in complex with cleaved DNA. *Nat. Struct. Biol.*, **7**, 792-799.
15. van der Woerd, M.J., Pelletier, J.J., Xu, S. and Friedman, A.M. (2001) Restriction enzyme BsoBI-DNA complex: a tunnel for recognition of degenerate DNA sequences and potential histidine catalysis. *Structure*, **9**, 133-144.

16. Etzkorn, C. and Horton, N.C. (2004) Mechanistic insights from the structures of HincII bound to cognate DNA cleaved from addition of Mg2+ and Mn2+. *J. Mol. Biol.*, **343**, 833-849.

17. Townson, S.A., Samuelson, J.C., Vanamee, E.S., Edwards, T.A., Escalante, C.R., Xu, S.-Y. and Aggarwal, A.K. (2004) Crystal structure of BstYI at 1.85 .ANG. resolution: A thermophilic restriction endonuclease with overlapping specificities to BamHI and BglII. *J. Mol. Biol.*, **338**, 725-733.

18. Hashimoto, H., Shimizu, T., Imasaki, T., Kato, M., Shichijo, N., Kita, K. and Sato, M. (2005) Crystal structures of type II restriction endonuclease EcoO109I and its complex with cognate DNA. *J Biol Chem*, **280**, 5605-5610.

19. Yang, Z., Horton, J.R., Maunus, R., Wilson, G.G., Roberts, R.J. and Cheng, X. (2005) Structure of HinP1I endonuclease reveals a striking similarity to the monomeric restriction enzyme MspI. *Nucleic Acids Res.*, **33**, 1892-1901.

20. Xu, Q.S., Roberts, R.J. and Guo, H.C. (2005) Two crystal forms of the restriction enzyme MspI-DNA complex show the same novel structure. *Protein Sci.*, **14**, 2590-2600.

21. Vanamee, E.S., Viadiu, H., Kucera, R., Dorner, L., Picone, S., Schildkraut, I. and Aggarwal, A.K. (2005) A view of consecutive binding events from structures of tetrameric endonuclease SfiI bound to DNA. *EMBO J.*, **24**, 4198-4208.

22. Bochtler, M., Szczepanowski, R.H., Tamulaitis, G., Grazulis, S., Czapinska, H., Manakova, E. and Siksnys, V. (2006) Nucleotide flips determine the specificity of the Ecl18kI restriction endonuclease. *EMBO J.*, **25**, 2219-2229.

23. Sokolowska, M., Kaus-Drobek, M., Czapinska, H., Tamulaitis, G., Szczepanowski, R.H., Urbanke, C., Siksnys, V. and Bochtler, M. (2007) Monomeric Restriction Endonuclease BcnI in the Apo Form and in an Asymmetric Complex with Target DNA. *J. Mol. Biol.*, **369**, 722-734.

24. Kaus-Drobek, M., Czapinska, H., Sokolowska, M., Tamulaitis, G., Szczepanowski, R.H., Urbanke, C., Siksnys, V. and Bochtler, M. (2007) Restriction endonuclease MvaI is a monomer that recognizes its target sequence asymmetrically. *Nucleic Acids Res.*, **35**, 2035-2046.

25. Sukackaite, R., Grazulis, S., Bochtler, M. and Siksnys, V. (2008) The recognition domain of the BpuJI restriction endonuclease in complex with cognate DNA at 1.3-A resolution. *J Mol Biol*, **378**, 1084-1093.

26. Lambert, A.R., Sussman, D., Shen, B., Maunus, R., Nix, J., Samuelson, J., Xu, S.Y. and Stoddard, B.L. (2008) Structures of the Rare-Cutting Restriction Endonuclease NotI Reveal a Unique Metal Binding Fold Involved in DNA Binding. *Structure*, **16**, 558-569.

27. Szczepanowski, R.H., Carpenter, M.A., Czapinska, H., Zaremba, M., Tamulaitis, G., Siksnys, V., Bhagwat, A.S. and Bochtler, M. (2008) Central base pair flipping and discrimination by PspGI. *Nucleic Acids Res*, **36**, 6109-6117.

28. Dunten, P.W., Little, E.J., Gregory, M.T., Manohar, V.M., Dalton, M., Hough, D., Bitinaite, J. and Horton, N.C. (2008) The structure of SgrAI bound to DNA; recognition of an 8 base pair target. *Nucleic Acids Res*, **36**, 5405-5416.

29. Golovenko, D., Manakova, E., Tamulaitiene, G., Grazulis, S. and Siksnys, V. (2009) Structural mechanisms for the 5'-CCWGG sequence recognition by the N- and C-terminal domains of EcoRII. *Nucleic Acids Res*, **37**, 6613-6624.

30. Watanabe, N., Takasaki, Y., Sato, C., Ando, S. and Tanaka, I. (2009) Structures of restriction endonuclease HindIII in complex with its cognate DNA and divalent cations. *Acta Crystallogr. D Biol. Crystallogr.*, **65**, 1326-1333.

31. Sokolowska, M., Czapinska, H. and Bochtler, M. (2009) Crystal structure of the beta beta alpha-Me type II restriction endonuclease Hpy99I with target DNA. *Nucleic Acids Res*, **37**, 3799-3810.

32. Mak, A.N., Lambert, A.R. and Stoddard, B.L. (2010) Folding, DNA Recognition, and Function of GIY-YIG Endonucleases: Crystal Structures of R.Eco29kI. *Structure*, **18**, 1321-1331.

33. Shen, B.W., Heiter, D.F., Chan, S.H., Wang, H., Xu, S.Y., Morgan, R.D., Wilson, G.G. and Stoddard, B.L. (2010) Unusual target site disruption by the rare-cutting HNH restriction endonuclease PacI. *Structure*, **18**, 734-743.

34. Firczuk, M., Wojciechowski, M., Czapinska, H. and Bochtler, M. (2011) DNA intercalation without flipping in the specific ThaI-DNA complex. *Nucleic Acids Res*, **39**, 744-754.

35. Manakova, E., Grazulis, S., Zaremba, M., Tamulaitiene, G., Golovenko, D. and Siksnys, V. (2012) Structural mechanisms of the degenerate sequence recognition by Bse634I restriction endonuclease. *Nucleic Acids Res*, **40**, 6741-6751.

36. Siwek, W., Czapinska, H., Bochtler, M., Bujnicki, J.M. and Skowronek, K. (2012) Crystal structure and mechanism of action of the N6-methyladenine-dependent type IIM restriction endonuclease R.DpnI. *Nucleic Acids Res*, **40**, 7563-7572.

39. Golovenko, D., Manakova, E., Zakrys, L., Zaremba, M., Sasnauskas, G., Grazulis, S. and Siksnys, V. (2014) Structural insight into the specificity of the B3 DNA-binding domains provided by the co-crystal structure of the C-terminal fragment of BfiI restriction enzyme. *Nucleic acids Res.*, **42**, 4113-4122.

40. Miyazono, K., Furuta, Y., Watanabe-Matsui, M., Miyakawa, T., Ito, T., Kobayashi, I. and Tanokura, M. (2014) A sequence-specific DNA glycosylase mediates restriction-modification in Pyrococcus abyssi. *Nat. Commun.*, **5**, 3178.
